# Impact of Korea’s emissions trading scheme on publicly traded firms

**DOI:** 10.1371/journal.pone.0285863

**Published:** 2023-05-24

**Authors:** Nyonho O., Daniela A. Miteva, Yehchan Lee

**Affiliations:** 1 Department of Agricultural, Environmental, and Development Economics, The Ohio State University, Columbus, Ohio, United States of America; 2 Isenberg School of Management, University of Massachusetts Amherst, Amherst, Massachusetts, United States of America; Shenzhen University, CHINA

## Abstract

Reducing fossil fuel energy consumption and greenhouse gas (GHG) emissions is vital to protecting life on the planet. Globally, emissions trading schemes are gaining traction as one way to curb emissions. However, the evidence of their effectiveness remains scarce. To address this gap, we examine the impact of Korea’s Emissions Trading Scheme (KETS), the first nationally mandated cap-and-trade program in East Asia to reduce GHG emissions, relative to its pre-existing command-and-control regulation called the Target Management System for Greenhouse Gases and Energy (TMS). Using panel data for publicly traded firms between 2011 and 2017, we apply a combination of panel data estimators and matching methods. We find that KETS did not significantly reduce emissions by firms but may have improved the aggregate efficiency in energy use in the energy and manufacturing sectors. Given the low levels of noncompliance with the first phase of the policy, it is likely that firms purchased permits and offsets or used previously banked permits to meet policy targets. Our work is one of the first efforts to understand the impact of KETS and the mechanisms underpinning its impact.

## Introduction

Reducing fossil fuel energy consumption and greenhouse gas (GHG) emissions, especially in energy-intensive countries, is important to mitigate climate change, protect ecosystems and people, and promote sustainability. Because of their greater compliance flexibility and cost-effectiveness, market-based instruments are often preferred to traditional command-and-control approaches to climate change mitigation [1, 2]; carbon taxes and emissions trading schemes (ETS), including baseline-and-credit systems and baseline-and-offset systems, are increasingly adopted in many locations. For example, as of 2022, 34 carbon emissions trading schemes (ETS) and 37 carbon taxes have been implemented or scheduled for implementation [3]. Despite the growing popularity of market-based instruments in climate change mitigation policy, it is still unclear whether they are more effective than command-and-control approaches in reducing carbon emissions.

South Korea’s climate change mitigation policies provide a unique setting to compare a market-based instrument, specifically a cap-and-trade system, to a command-and-control approach in terms of environmental performance. To increase the country’s sustainability and contribute to international climate change mitigation efforts, the South Korean government implemented the Target Management System for Greenhouse Gases and Energy (TMS), a command-and-control approach, in 2010, followed by Korea’s Emissions Trading Scheme (KETS), a cap-and-trade scheme, in 2015. Both regulations set reduction targets based on the firm’s historical emissions and production levels. Also, because the TMS and the KETS utilize the same measure, verification, and reporting systems, comparisons between the two are credible; the TMS provides baseline data for evaluating the KETS.

We focus on the impact of the ETS in a non-European country using detailed firm- and facility-level emissions data in South Korea. South Korea is the fourteenth-largest economy in terms of Real GDP, the seventh-largest CO_2_ emitter, and the fifteenth-largest energy consumer per capita [4]. The KETS is the first nationally mandated carbon emissions trading scheme (ETS) in East Asia, boasting the third-largest carbon market in the world [5]. There exist only a few non-European countries that have established national ETSs, namely New Zealand (2008-), Kazakhstan (2013-), South Korea (2015-), and China (2021-). The evidence on the impact of the ETS in these countries is still limited: Discussion of the New Zealand ETS focuses on the inclusion of forestry [6, 7], market behavior [8], and design features [9, 10]. The evaluation of China’s national ETS is yet to come due to the delay of its operations. Existing literature exploits China’s regional ETS pilots to estimate the causal impact [11–13]. However, most studies use provincial energy consumption data to compute carbon emissions due to the lack of firm-level emissions data. Due to limited data availability on carbon emissions for non-participating firms and pre-ETS periods, only a handful of studies exploited firm- or facility-level data to estimate the impact of the ETS on carbon emissions based in Europe [14–18].

The impact of the KETS remains understudied, with work focusing on a specific industry, such as petrochemical [19], steel [20], and power plants [21, 22], from an efficiency standpoint. Three prior studies assess the overall effectiveness of the KETS. Using a computable general equilibrium model with 525 firms initially targeted by the KETS, Choi et al. [23] showed that the KETS could reduce GHG emissions by 2.56%, with GDP and industry outputs decreasing by 0.41% and 0.54%, respectively. Using firm-level data from the manufacturing, building, and power industries for 2013–2017, Jun et al. [24] found that the first phase of the KETS improved the carbon intensity in the manufacturing and building sectors but not in the power sector. However, the first difference approach used in the study raises some concerns about serial correlation and endogeneity stemming from simultaneity in the estimation. Using a panel dataset and a duration curve model, Oh et al. [25] found small impacts of KETS on improving the energy intensity through changes in energy sources in the manufacturing sector but no effects in the power generation sector or overall emissions. However, neither study differentiates between publicly traded and private companies.

Our work evaluates the overall environmental performance of the KETS relative to the TMS in terms of GHG emission and energy consumption for publicly traded firms. Using panel data that allow us to differentiate between facilities and firms in the energy and manufacturing sectors for 2011–2017, we conduct careful and rigorous impact evaluation on the impact of the first phase of the KETS on total emissions and energy intensity for publicly traded firms. In addition to rigorous statistical methods—two-way fixed effects, matching, and synthetic difference-in-differences models, we build on previous studies by focusing on publicly traded companies and differentiating by the level of reporting. Publicly traded companies are important as they tend to invest more in R&D than similar private firms, potentially due to lower costs of capital [26]; they may also respond to demand shocks more quickly [27]. Therefore, given the short duration of the first phase of KETS, we expect publicly traded companies to exhibit the most changes if the program design is effective. Consistent with previous studies, we find that the first phase of the KETS did not significantly reduce carbon emissions but improved efficiency in energy use. We did not find any statistically significant differences based on the level of reporting (facility vs. firm). Overall, we contribute to the growing body of work that offers insights into the impact of the initial phase of the KETS, with important implications for the KETS design.

## Study area

### Korea’s greenhouse gas reduction policies

In the 2009 United Nations Climate Change Conference, South Korea declared to reduce greenhouse gas (GHG) emissions by 30% relative to business-as-usual level by 2020 [28]. The Framework Act on Low Carbon and Green Growth in 2010 introduced two policies to achieve the reduction of carbon emissions: (1) Target Management System for Greenhouse Gas and Energy (TMS) and (2) Korea’s market-based Emissions Trading Scheme (KETS). Participation in both is based on whether the average of past 3 years’ emissions exceed a pre-determined threshold.

The main difference between the two programs is that firms under the TMS must reduce the emissions through internal efforts during the implementation period, while firms participating in the KETS have outside options, such as purchasing permits from other firms or borrowing permits from the future allocations. Depending on the (expected) price levels of the permits under the KETS, the participating firms may decide whether to offset the emissions by market transactions or exert efforts to reduce the emissions.

### Target management system (TMS)

Based on Article 42 of the Framework Act on Low Carbon and Green Growth, the TMS came into effect in April 2010. Targeted firms are designated as controlled entities if the annual average amount of greenhouse gas emissions or energy consumption in the last three years is over a specified threshold (Table 1). Specifically, a firm receives a firm-level designation if the annual aggregated amount of GHG emissions or energy consumption across the firm’s all facilities exceeds the appointed amount at the firm-level (Korea Environment Institute 2012). If one of the firm’s facilities emits GHG or consumes more energy than the specified facility-level amount, but the total amount of the entire firm is below the firm-level threshold, then the designation is made at the facility-level [28]. The thresholds to identify the controlled entities decrease over time to include more firms under control while allowing more time for small firms to prepare for the regulation [28]. Note that in this article, “facility” means a place of business where business activities, such as the production of goods and services, occur within physical boundaries. A "firm” means a collection of business places belonging to the same organization.

**Table 1:** Criteria for identifying targeted firms by TMS (Republic of Korea 2010).

	Until December 3 ^1s^ t 2011		From January ^1s^t 2012		From January ^1s^t 2014	
Reporting level	Firm	Facility	Firm	Facility	Firm	Facility
Greenhouse Gas Emissions (ton CO_2_eq)	125,000	25,000	87,500	20,000	50,000	15,000
Fossil Energy Consumption (terajoule)	500	100	350	90	200	80

Once a responsible ministry makes the designation, a designated firm must report GHG emissions and energy consumption, which a third-party contractor must verify. The targets of reducing GHG emissions and energy consumption are set through negotiation processes between the responsible ministry and the designated firm by the end of September next year from the year of the designation. However, the reduction target of GHG must be consistent with the national goal of reducing GHG by 30% relative to the BAU level by 2020 (or 37% by 2030). Then, the designated firm must submit plans to achieve the targets by the end of December of the following year from the year of the designation. The designated firm implements the management plans in the next year, followed by submitting a performance report of the reduction plan. A responsible ministry may issue an improvement order if there is poor implementation performance or reporting misrepresentation. If the firm fails to address the improvement order, the Ministry of Environment may issue a fine that ranges from KRW 3 million to 10 million (approximately USD 2,500 to 8,333 assuming the currency’s conversion rate is USD 1.00 = KRW 1,200), depending on the degree of non-compliance. The fines are not large: $2500 is less than the monthly income of an average person in Korea.

In addition, firms are incentivized to reduce emissions before being regulated by the TMS. The voluntary reductions before the TMS are counted towards their reduction amounts during the implementation period, although the early reduction amounts cannot exceed 1% of the total emissions. Moreover, designated firms may participate in GHG reduction projects outside the firm’s boundaries to offset the reduction target.

### Korea’s emissions trading scheme (KETS)

The legislation of the Framework Act on Low Carbon, Green Growth in 2010 provided a legal basis for Korea’s Emissions Trading Scheme. In 2012, the Parliament of Korea approved the Act on the Allocation and Trading of Greenhouse-gas Emission Permits, calling for the introduction of the KETS in January 2015 (Korea Environment Institute 2015). As of 2014, the firm becomes no longer subject to the TMS and automatically enrolled in the KETS if a firm annually has emitted GHG over 125,000 tons CO_2_eq at the firm-level on average over the last three years (i.e., 2011–2013) or has a facility annually releasing GHG more than 25,000 tons CO_2_eq on average in the last three years. Also, firms may voluntarily participate in the KETS.

The first phase of the KETS took place from 2015 to 2017; the second phase was implemented in 2018, running through 2020. The scheme initially targeted 525 firms responsible for 60% of the country’s total greenhouse gas emissions. In 2015, 44 entities were added to the scheme; 34 entities were additionally included in 2016. The total GHG allowance for the first phase to the 525 firms was 1,622.6 million tons CO_2_eq: 545.9 million tons CO_2_eq in 2015, 535.1 million tons CO_2_eq in 2016, and 541.6 million tons CO_2_eq in 2017. However, after considering new entrants, the adjustment or the cancellation of the allowance amounts, and the firm’s early GHG reduction, the total GHG allowance became 1,686.3 million tons CO_2_eq. Each year the annual allowance amount increased by 4%: 540.1 million tons CO_2_eq in 2015, 560.7 million tons CO_2_eq in 2016, and 585.5 million tons CO_2_eq. The actual GHG emissions during the first phase were 1,668.9 million tons CO_2_eq, which is 1% less than the total GHG allowance.

The participating firms must submit an emissions report and obtain emission permits according to the actual emissions. In case of holding an insufficient number of permits, borrowing from other years in the same phase, for example between 2015 and 2017 in the first phase, is allowed; saving extra permits for the next phase is also accepted. In addition to purchasing permits in the trading market, the participating firms may operate local Clean Development Mechanism (CDM) projects or invest in similar projects abroad to earn emission credits. The penalty for holding insufficient permits is the extra amount of emissions multiplied by (at a maximum) triple the annual average price per ton CO_2_eq in the trading market. However, the penalty price per tonCO_2_eq shall not exceed KRW 100,000 (approximately USD 83.33) (Korea Environment Institute 2015). Only one firm in 2015 and two firms in 2017 failed to hold enough amounts of the permits [29].

## Methods

Although both TMS and KETS share the same national goal in GHG emissions reductions, the KETS allows firms more flexibility through banking, offsets, and trading emission permits across years and among participating firms [30]. This implies that firms under the KETS may have an incentive to *overly* achieve the goal as they can sell surplus permits in the market. This is the hypothesis we test in this paper.

### Average treatment effects

We focus on the performance of the cap-and-trade policy relative to the TMS. Our treatment group consists of firms that were targeted under the TMS until 2014 and entered the trading scheme in 2015; the control group is comprised of firms that have been subject to the TMS and never participated in the KETS. That is, Groups 1, 2, 3, and 7 comprise the treatment group participating in the KETS in 2015; Groups 13, 14, 15, and 16 comprise the control group that had been regulated by the TMS but never entered the KETS (See Table 2).

**Table 2:** Treatment (KETS) and control (TMS) groups based on the year of enrollment.

Group	Target Management (TMS) (Entering year)	Emissions Trading (KETS) (Entering year)	Reporting level: Firm	Reporting level: Facility	Total
1	2011	2015	86	57	143
2	2012	2015	1	21	22
3	2013	2015	0	2	2
4	2013	2017	0	2	2
5	2013	2018	0	1	1
6	2013	2019	0	1	1
7	2014	2015	1	0	1
8	2014	2016	0	6	6
9	2014	2017	0	2	2
10	2014	2018	0	3	3
11	2015	2017	0	1	1
12	2016	2018	0	2	2
13	2011	N/A	0	2	2
14	2012	N/A	0	4	4
15	2013	N/A	1	4	5
16	2014	N/A	1	25	26
17	2016	N/A	0	6	6
Total					229

*Note*: In group 1, 2, and 6, there are firms switching their reporting levels over the years. Therefore, the total number of firms in groups 1, 2, and 6 is different from the sum of company-level reporting firms and facility-level reporting firms.

To estimate the impact of the KETS, we employ difference-in-differences (DID) and triple difference methods. The latter allows us to examine the role of the level of reporting. Throughout the analysis, we only consider two periods: before and after the introduction of the KETS. There are 2 reasons for collapsing the data to before and after periods: (1) the small sample size and (2) the ability of firms to bank permits and borrow from future permits and allocations within the first phrase, resulting in serial correlation.

The DID approach is pivoted on the parallel trends assumption. However, differences in covariate distributions may be driving the results. Therefore, to ensure the control and treatment groups have overlapping covariate distributions at the baseline, we supplement the panel data analysis with matching. As a robustness check, we perform a synthetic difference-indifferences estimation.

We consider the following DID model:

$$y_{i,t}=c+\alpha_{1}T_{t}+\alpha_{2}T_{t}\cdot D_{i}+{\bar{X}}_{i0}^{\prime }\beta +\delta_{i}+\epsilon_{i,t}$$ (1)

where *i* indicates a firm, *t* represents a year, *y*_*i*,*t*_ is an outcome variable—we use the log of GHG emissions and the log of energy consumption. *T*_*t*_ is a dummy variable indicating before (= 0) and after (= 1) the introduction of the KETS, *D*_*i*_ is a treatment status dummy (= 1 if treated, 0 otherwise). ${\bar{X}}_{i,o}$ is a vector of a firm’s characteristics prior to the introduction of KETS; δ_*i*_ is a firm-specific fixed effect; ϵ_*i*,*t*_ is an error term assumed to be independent of controlled variables. The coefficient of interest is α_2_ which captures the average effect of the KETS on the changes in GHG emissions or energy consumption over time conditional on covariates, ${\bar{X}}_{i,0}$. Note that because of individual fixed effects, we cannot estimate a coefficient on Di by itself; the model allows us to estimate only the interaction with the time variable α_2_.

The covariates we use are the log of average sales before 2015, average return on assets before 2015, and average debt ratio before 2015. These are commonly used in studies of firms’ environmental and financial performance [31, 32]. The log of sales controls for the size of a firm and serves as a proxy for the firm’s outputs [33]; the return on assets (ROA) indicates for the profitability of a firm, and the debt ratio—the level of a firm’s financial distress. The ROA is measured by profits divided by average total assets; the debt ratio is computed by total liabilities divided by total assets.

### Additional statistical considerations

#### Parallel trends assumption

To examine the support for the parallel trend assumption, we estimate the following specification:

$$y_{i,t}=\gamma_{0}+\lambda_{t}+\gamma_{1}year_{2011}\cdot D_{i}+\gamma_{2}\;year_{2012}\cdot D_{i}+\gamma_{3}\;year_{2013}\cdot D_{i}+\gamma_{4}\;year_{2015}\cdot D_{i}+\gamma_{5}\;year_{2016}\cdot D_{i}+\gamma_{6}\;year_{2017}\cdot D_{i}+{\bar{X}}_{i,0}\phi +\mu_{i,t}$$ (2)

where time *t* varies from 2011 to 2017, λ_*t*_ represents year-fixed effects, and *year*_*t*_ is a dummy variable for each time *t*, *year*_*t*_*·D*_*i*_ is an interaction term between a year dummy and a treatment status dummy, and ${\bar{X}}_{i,t}$ is a vector of firm’s characteristics. *year*_*2014*_ is omitted to serve as a baseline year comparing before and after the treatment periods. The coefficients of interest in this specification are γ_1_, γ_2_, γ_3_. Coefficients that are statistically indistinguishable from 0 lend support to the parallel trends assumption.

### Preprocessing the sample

Even if the pre-treatment trends are parallel, conditional on the covariates, concerns may remain that the differences in the outcomes between the treatment and control groups are due to their characteristics and not necessarily the KETS [34]. To ensure covariate overlap between the treatment and control groups, we first employ nearest neighbor covariate matching with replacement and a Mahalanobis distance metric, with trimming based on the propensity score, and calipers equal to 0.05. The approach helps us exclude observations with very different characteristics and ensure the covariate overlap of the treatment and control groups.

We perform matching by first estimating a logistics regression using the *average GHG before 2015*, *average energy before 2015*, *average sales before 2015*, *average ROA before 2015*, *and average debt ratio before 2015* to obtain the estimated linearized propensity score, which is the predicted probability of KETS participation. We include the annual average of greenhouse gas emissions and energy consumption before introducing the KETS, as those are critical determinants in KETS participation. We then use covariate matching, including the estimated propensity scores within calipers on the common support. This approach performs the best with our data.

Using the matched sample, we re-estimate Eq (1) using frequency weights to account for the fact that some observations in the control group are used more than once in the matching. It has been suggested that the combination of matching and fixed effects panel data estimators can significantly reduce bias and approximate experimental estimates [35].

### Treatment heterogeneity

The policy may have affected firms differently depending on whether they report emissions at the firm or facility levels. To test for heterogeneity in the impact based on the reporting level, we estimate the following triple difference model:

$$y_{i,t}=\kappa_{0}+\kappa_{1}T_{t}+\kappa_{2}D_{i}+\kappa_{3}F_{i}+\kappa_{4}T_{t}\cdot D_{i}+\kappa_{5}T_{t}\cdot F_{i}+\kappa_{6}D_{i}\cdot F_{i}+\kappa_{7}T_{t}\cdot D_{i}\cdot F_{i}+{\bar{X}}_{i,0}\psi +\delta_{i}+\nu_{i,t}$$ (3)

where *F*_*i*_ is a categorical variable indicating firm *i*’s reporting level (= 1 if reporting at firm-level, or = 2 at facility-level), and the rest is the same as in Eq (1). The coefficient of interest is κ_7_. Because of our relatively small sample size and limited variation in the reporting level, we cannot perform the triple difference estimation after pre-processing the sample via matching.

### Robustness checks

#### Synthetic difference-in-differences

We examine the performance of each treatment group (based on the year of enrollment in Table 2) relative to the control groups. Specifically, we compare Group 1 with Group 13, Group 2 with Group 14, and Group 3 with Group 15. Also, we collectively compare Groups 1, 2, and 3 with Groups 13, 14, and 15 (Table 2). We leave out Group 7 and Group 16 as they started reporting in 2014, which makes it impossible to ensure parallel trends before KETS was introduced in 2015. Note that Group 1 consists of the largest emitters in the country, and Group 3 has the smallest emitters among the treated. (See Table 2 to see the group composition and the number of firms for each group.)

To examine the impact of the enrollment time, we use synthetic difference-in-differences estimation [36]. Specifically, the method ensures support for the pre-treatment parallel trends by reweighting treatment and control observations based on how similar their covariates are: Observations with low similarity are given little weight, whereas very similar observations—higher weights. The covariates we use for the pre-treatment parallel trends are the same as for Eq (1)—log of sales, ROA, and Debt Ratio.

Estimating synthetic difference-in-differences conditioning on covariates is a two-step procedure. The first step is to obtain residual after running the following regression:

$$y_{it}^{res}=y_{it}-{\bar{X}}_{i0}\hat{\beta }$$ (4)

where $\hat{\beta }$ is determined by regression and ${\bar{X}}_{i0}$ contains the covariates used in the above equations. The second step estimates the following equation:

$$(\hat{\tau },\hat{\mu },\hat{\alpha },\hat{\gamma })=\underset{\tau ,\mu ,\alpha ,\gamma }{\mathrm{argmin}}\left\{\sum_{i=1}^{N}\sum_{t=1}^{T}{\left(y_{it}^{res}-\mu -\alpha_{i}-\gamma_{t}-D_{it}\tau \right)}^{2}{\hat{\omega }}_{i}{\hat{\lambda }}_{t}\right\}$$ (5)

where *μ* is a constant, *α*_*i*_ is a unit-fixed effect, *γ*_*t*_ is a time-fixed effect, *D*_*it*_ is a dummy equal to 1 when treated by the KETS and zero otherwise, and ${\hat{\omega }}_{i}$ and ${\hat{\lambda }}_{t}$ are optimally chosen weights to ensure pre-treatment parallel trends. $\hat{\tau }$ estimates an average treatment on the treated effect of the KETS. Standard errors are estimated by block bootstrap.

### Potential alternative approaches

Since there is a threshold to determine the participation of the KETS, one may suggest conducting a regression discontinuity (RD) analysis. That is, under the assumption that firms on both sides of the threshold are similar to each other, one can estimate the average treatment effect of the KETS by examining a subset of firms emitting GHGs just below and above 125,000 tons CO2eq per year on average before 2015 (or 25,000 tons CO2eq in the case of a facility). The limited number of firms in our sample, however, does not allow for an RD estimation. For example, the number of firms emitting GHGs between 120,000 and 130,000 tons CO2eq per year on average before 2015 is only five; all of these firms are in the treatment group. Also, the number of firms reporting at the facility-level whose annual average emission ranges from 20,000 to 30,000 tons CO2eq before 2015 is 27. Together, we only have 32 firms.

### Data

The Korean National Greenhouse Gas Management System provides statements of greenhouse gas emissions and energy consumption submitted by regulated firms. The statement includes a firm’s name, year of designation, industry, greenhouse gas emissions, energy consumption, and the government ministry responsible for its designation. The longest span of the greenhouse gas emissions data is from 2011 to 2017, and the shortest is from 2016 to 2017. Unfortunately, the firm’s greenhouse gas emission levels are only available after the regulations targeted it. It also should be noted that the TMS and the KETS target five sectors: energy, manufacturing, buildings, transportation, and public sectors [29]. Among these sectors, we focus on the energy and manufacturing sectors (integrated energy supply(industrial), mining, food and beverages, textiles, wood, pulp, oil refining, petrochemicals, glass, ceramics, cement, steel, nonferrous metals, machinery, semiconductors, displays, electrical and electronics, automobiles, shipbuilding, telecommunications) first because these two sectors take up the majority of the GHG emissions among the regulated sectors. Further, it is challenging to obtain firm-level characteristics for buildings and public sectors, which consist of buildings, regional governments, and state-owned firms.

We focus on only the publicly traded firms among the regulated firms. Firms traded in Korea’s stock markets must submit accounting and business reports to the Financial Supervisory Service, which oversees the entire Korean financial markets. The submitted reports are open to the public through the Financial Supervisory Service. Combining the emission statements and financial reports, our dataset contains 229 firms. However, since we only consider firms participated in 2015 and those who never participated, our treatment and control groups contain 205 firms in total.

**Fig 1:**
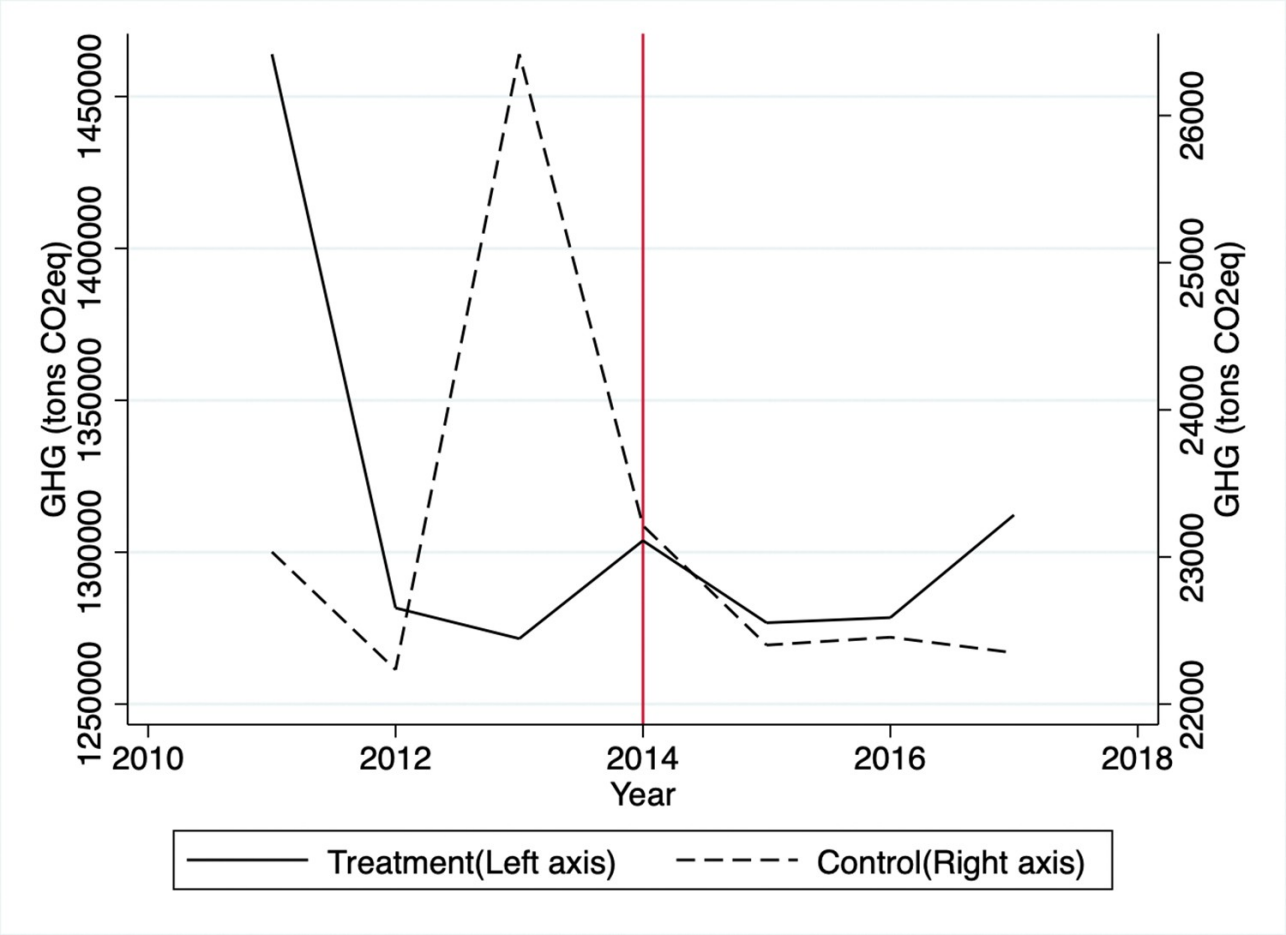
Average GHG emissions.

**Fig 2:**
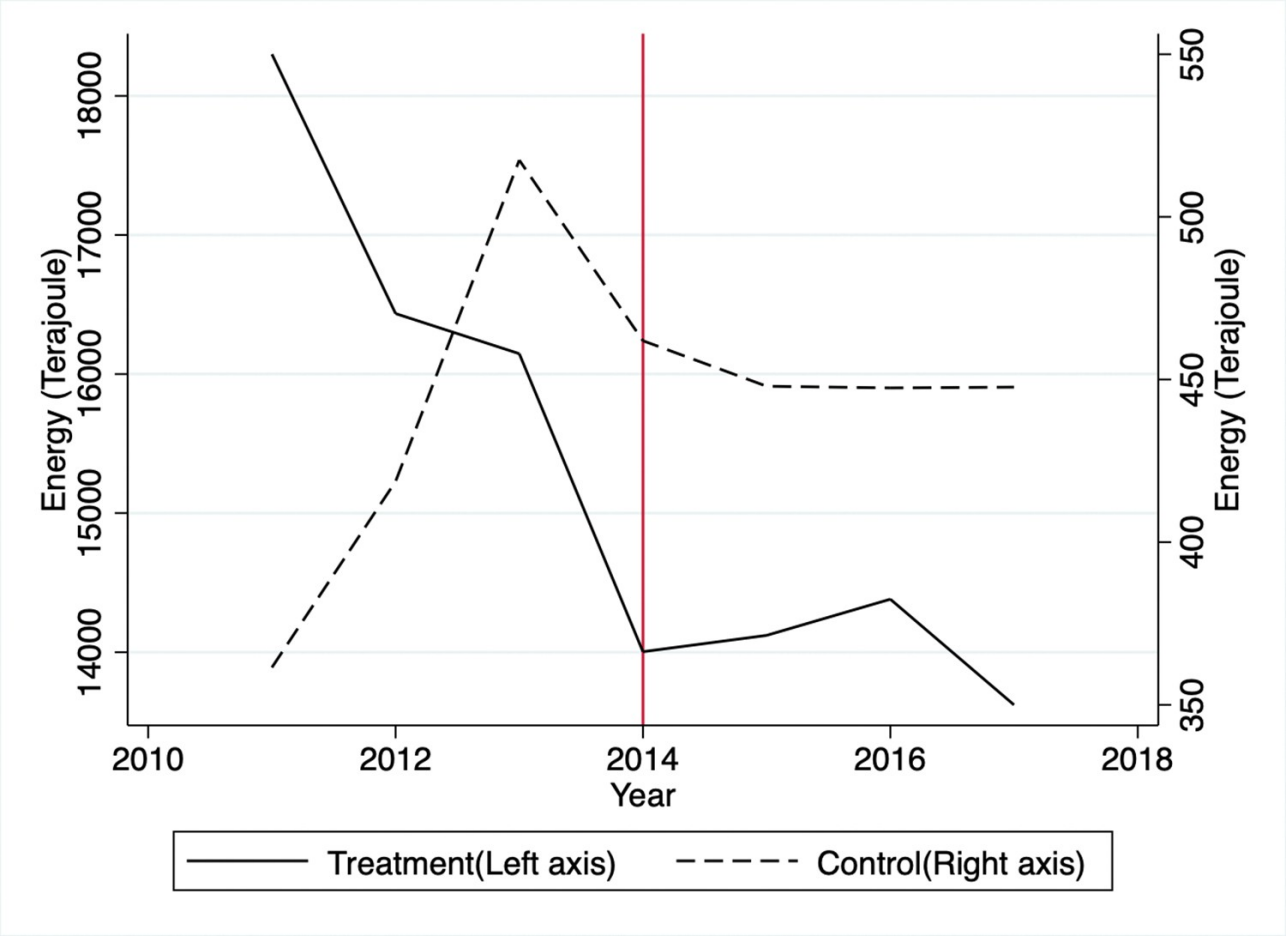
Average energy consumption.

Based on the raw data for the treatment group, GHG emissions sharply dropped from 2011 to 2012 and fluctuated for the rest of the period (Fig 1). A sharp decrease is due to the average GHG emission levels of group 1 being much higher than the rest of the treated groups. Note that the TMS targeted heavy emitters first in 2011 and decreased its targeting thresholds to include relatively smaller emitters in later years. In the control group, the GHG emissions soared from 2012 to 2013, plummeted from 2013 to 2015, and leveled out. The energy consumption for the treatment group decreased sharply by 2014 and fluctuated a bit until 2017. Mirroring the GHG trend, the control group’s energy consumption rose rapidly from 2011 to 2013, fell from 2013 to 2015, and leveled off after that (Fig 2).

Table 3 presents the mean of outcome variables and other covariates across all the years and the firms in the treatment and control groups. As can be seen, the treatment and control groups are considerably different on average, particularly in terms of GHG emissions, energy consumption, sales, and debt ratio; normalized differences of such covariates are 0.298, 0.372, 0.235, and 0.204, respectively. The large differences in the distributions of these covariates across the treatment and control groups might introduce bias [34]. In general, the treated firms are larger than the control firms in terms of size and emissions. However, the profitability, measured by ROA, of the treated and controlled firms is the same on average between the two groups. Also, given the higher average debt ratio, the treated firms are more likely to face higher costs to raise capital, which might discourage the firms from investing in the abatement of GHG emissions.

**Table 3:** Mean comparisons of treatment and control groups.

	Control	Treatment	Normalized difference^a^
Greenhouse gas	22,847.50	1,309,459.98	0.298
(tons CO_2_eq)	(17,401.04)	(6,097,308.03)	
Energy	453.36	15,217.52	0.372
(terajoules)	(354.69)	(56,119.30)	
Sales	2,848,550,185.92	6,745,739,979.00	0.235
(KRW 1,000)	(11,310,829,048.23)	(20,510,140,279.32)	
Return on assets	2.23	2.23	0.0
	(7.12)	(6.65)	
Debt ratio	0.46	0.51	0.204
	(0.20)	(0.20)	
Num. firms	37	168	

Standard deviations in parentheses

^a^ the following formula calculates a Normalized difference: $\frac{│{\bar{x}}_{treat}-{\bar{x}}_{control}│}{\sqrt{s_{treat}^{2}+s_{control}^{2}/2}}$, where s^2^ indicates standard deviations and $\bar{x}$ is the mean for a covariate for the treatment and control groups, respectively.

## Results

### Average impact of KETS

Using the unmatched sample, we find that on average KETS increased the GHG emissions and energy consumption of the enrolled firms by approximately 6% (Table 4 Panel A). We find the support for the parallel trends assumption underpinning the unmatched DID model (S1 Table).

**Table 4:** Difference-in-differences estimates.

	(1)	(2)
	Ln(GHG)	Ln(Energy)
*A*. *Before Matching*		
T	-0.0869***	-0.0790***
	(0.0289)	(0.0256)
T X D	0.0661*	0.0568*
	(0.0340)	(0.0316)
Observations	1314	1314
Num. Firms	205	205
Control	Yes	Yes
FE	Yes	Yes
*B*. *After Matching*		
T	-0.0635	-0.0559
	(0.0522)	(0.0447)
T X D	0.0322	0.00696
	(0.0693)	(0.0667)
Observations	346	346
Num. Firms	49	49
Control	Yes	Yes
FE	Yes	Yes

Standard errors in parentheses

* *p* < .1

** *p* < .05

*** *p* < .01

The DID estimation controlling for the differences in covariate distributions results in positive but insignificant coefficients (Table 4 Panel B). However, while matching greatly reduced the normalized differences between the treatment and control groups (S2 Table), it also significantly reduced the sample size: The number of firms in the control group decreased from 37 to 18. The number of firms in the treatment group dropped from 168 to 31.

### Heterogeneity of impacts

#### Triple-difference estimator

To examine the impact of the level of reporting, the coefficient on *Treated X KETS X Facility* in Eq (3) is of interest. The results based on the unmatched sample indicate a positive and significant at the 10% level increase in GHG emissions and energy consumption by about 15% for firms reporting at the facility-level relative to the firm-level reporting. Unfortunately, with the matched sample, we cannot apply a triple difference estimator, as we lose most of our observations through pre-processing the data (Table 5).

**Table 5:** Triple difference estimates without matching.

	(1)	(2)
	Ln(GHG)	Ln(Energy)
T	0.0185	0.0207
	(0.0718)	(0.0717)
T X D	-0.0567	-0.0634
	(0.0758)	(0.0757)
T X F	-0.112	-0.106
	(0.0778)	(0.0764)
T X D X F	0.149*	0.150*
	(0.0857)	(0.0849)
Observations	1314	1314
Num. Firms	205	205
FE	Yes	Yes
Control	Yes	Yes

Standard errors in parentheses

* *p* < .1, ** *p* < .05, *** *p* < .01

#### Synthetic difference-in-differences estimator

We find heterogeneity in the impact of KETS based on when a firm became enrolled. Specifically, Group 1, the largest emitter (treated), emitted 18% more GHGs than Group 13 (control), while the differences between the other treatment and control groups is statistically indistinguishable from 0 (Table 6). Unsurprisingly, the aggregate average treatment effect is positive and statistically significant, due to the increase in GHG emissions by Group 1. We find similar results for energy consumption.

**Table 6:** Synthetic difference-in-differences estimates.

	(1)	(2)	(3)	(4)
	Ln(GHG)	Ln(Energy)	Ln(Intensity of GHG)	Ln(Intensity of Energy)
*Group 1 vs*. *Group 13*				
ATT	0.179***	0.145***	0.118*	0.107
	(0.0428)	(0.0312)	(0.0620)	(0.0763)
*Group 2 vs*. *Group 14*				
ATT	0.0558	0.0776	-0.145	-0.155
	(0.112)	(0.115)	(0.235)	(0.218)
*Group 3 vs*. *Group 15*				
ATT	-0.172	-0.203	0.0706	0.0222
	(0.174)	(0.170)	(0.183)	(0.186)
*Group 1*,*2*,*3 vs*. *13*,*14*,*15*				
ATT	0.108**	0.0715*	0.0239	0.00536
	(0.0456)	(0.0392)	(0.0958)	(0.0882)

Standard errors in parentheses

* *p* < .1

** *p* < .05

*** *p* < .01

### Examining potential threats to the identification

#### Dodging the KETS

**Fig 3:**
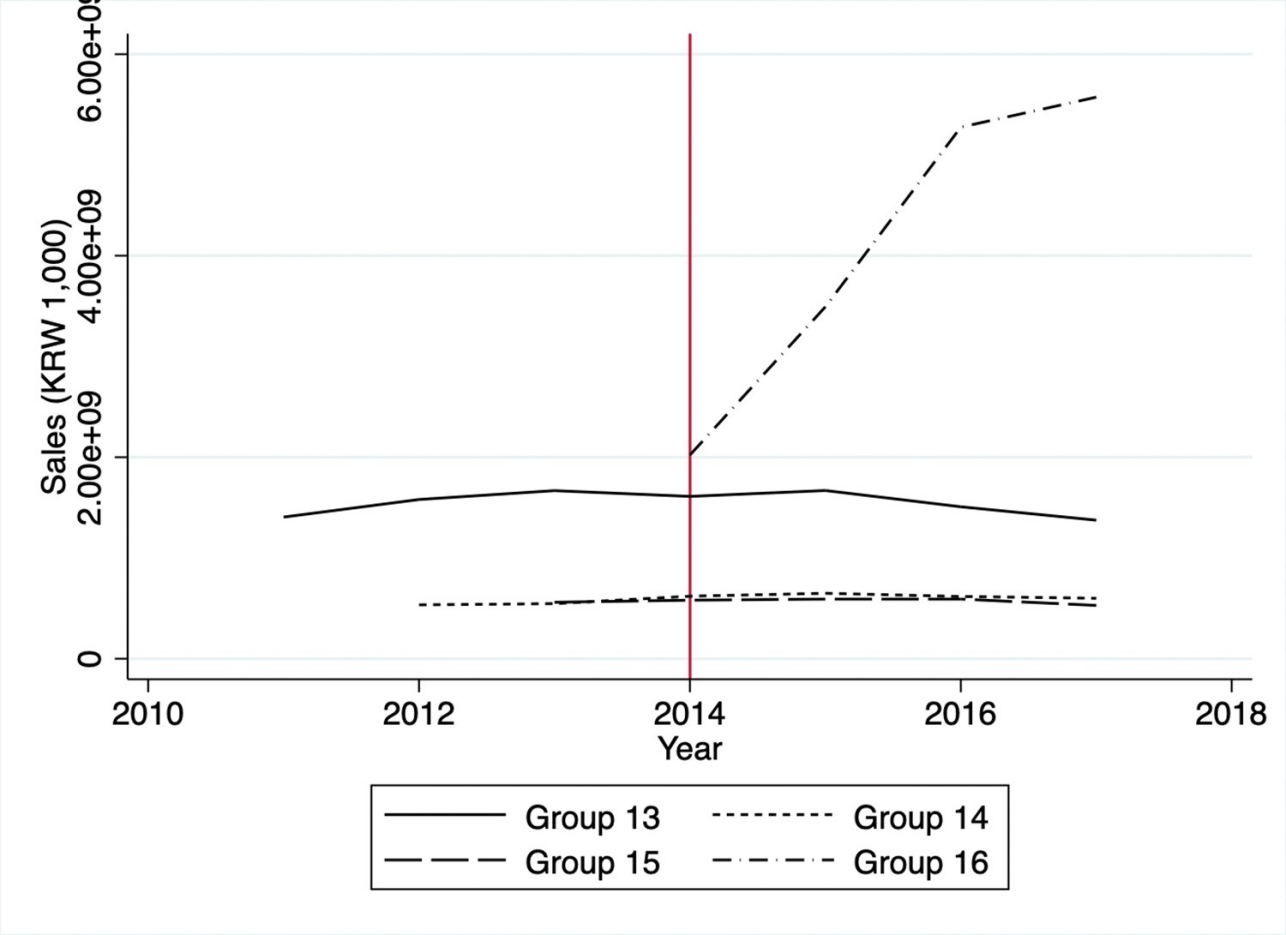
Average sales of control group.

**Fig 4:**
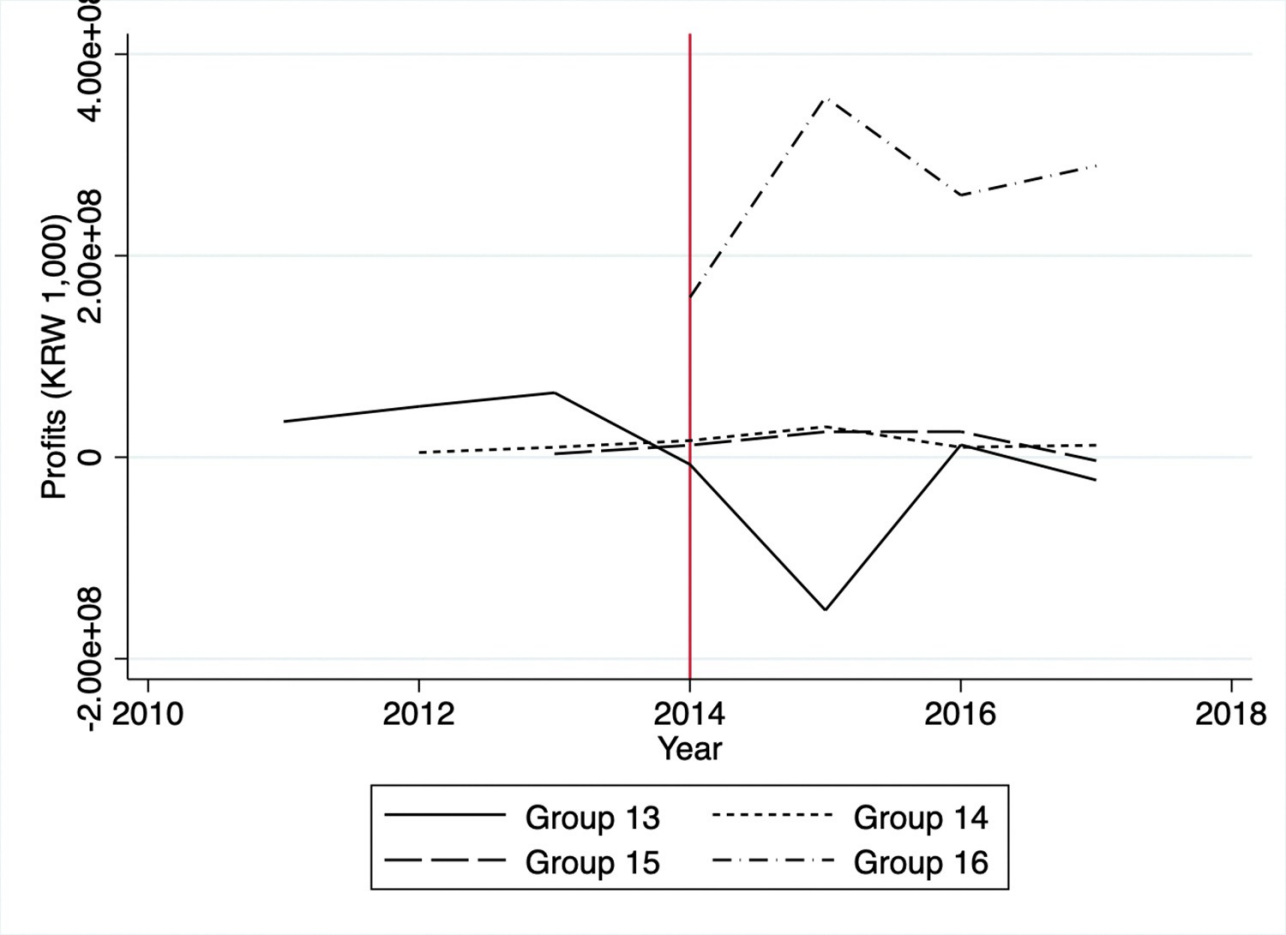
Average profits of control group.

**Fig 5:**
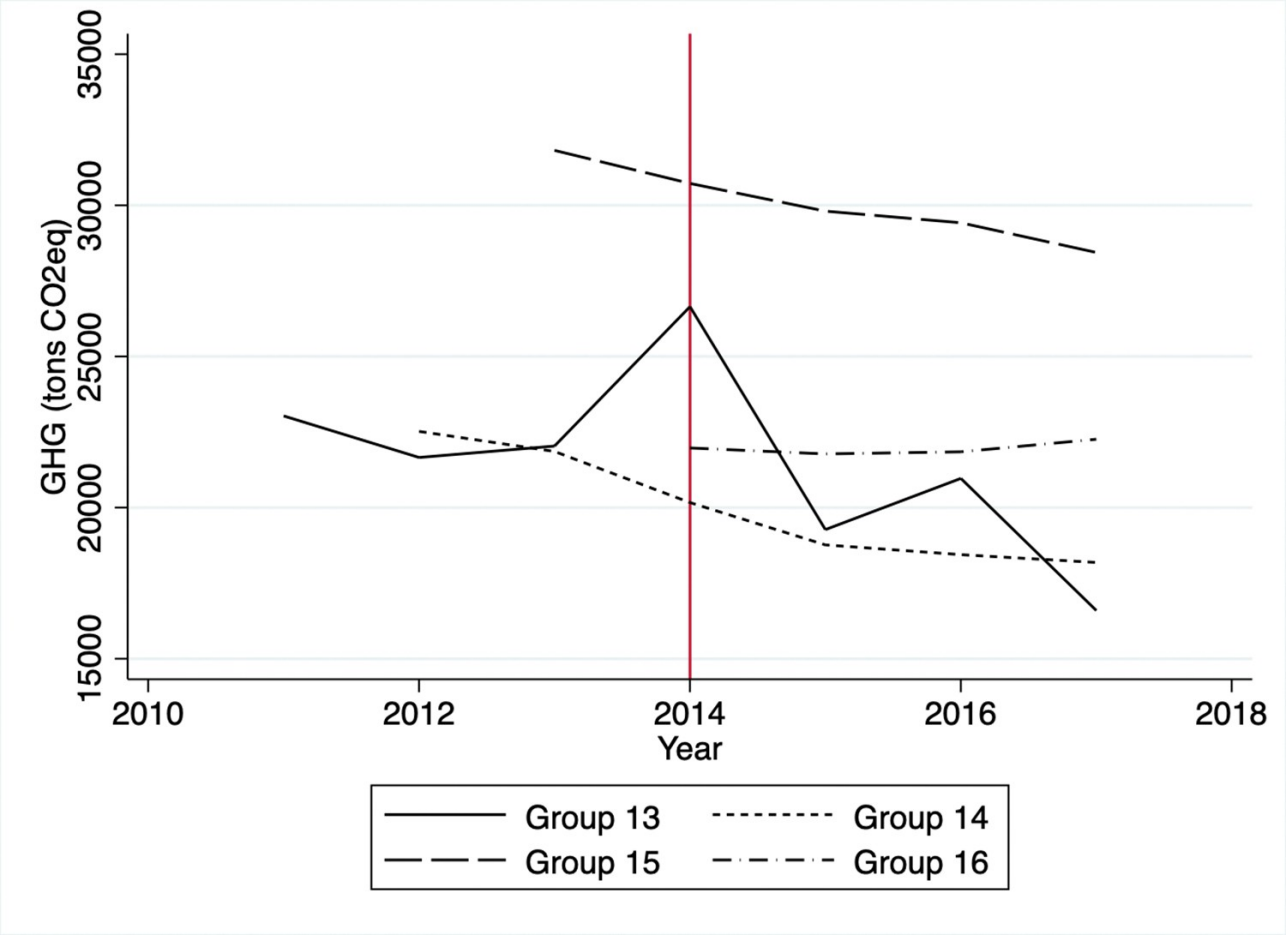
Average GHG emissions of control group.

**Fig 6:**
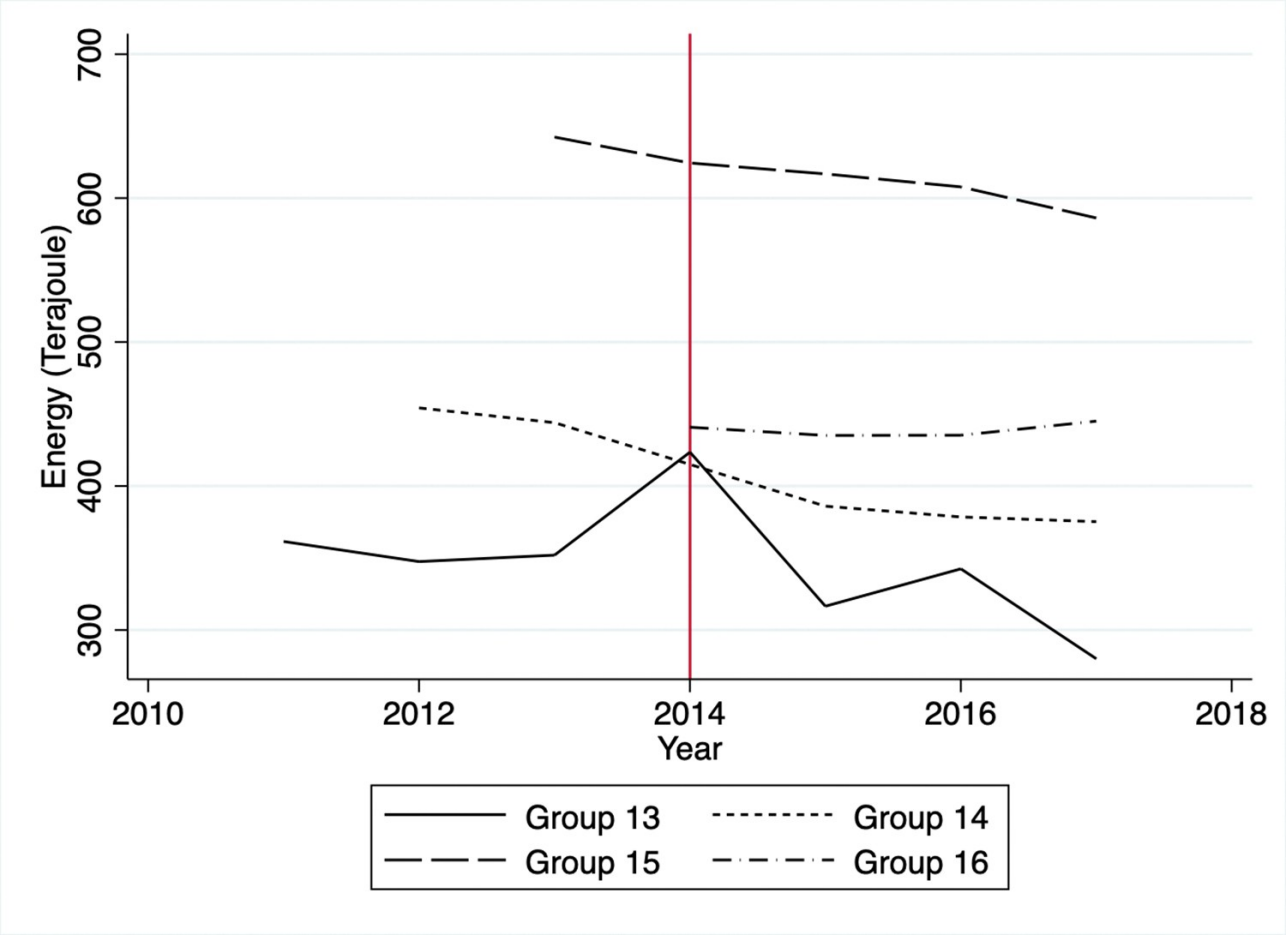
Average energy consumption of control group.

A potential threat to the identification is that firms might avoid automatic enrollment in the KETS by limiting outputs to keep GHG emissions just below the threshold. Specifically, one can suspect that firms in the control group might have been able to remain outside the KETS by reducing outputs and GHG emissions. This situation would happen if the costs of reducing GHG emissions by entering the KETS exceeded the benefits of producing more goods. Since we do not have information regarding individual firm’s outputs, we can only proxy the outputs via the firm’s sales or profits. Visual inspection of Figs 3 and 4 reveals no significant decreases in sales or profits in the controlled firms between 2011 and 2014. We observe mostly flat trends in sales and profits except for group 16, which started reporting emission activities in 2014, while GHG emissions and energy consumption had decreased overall (Figs 5 and 6).

#### Change in the intensity of GHG emissions/energy consumption

Although we find primarily positive impacts of the KETS on the GHG emission and energy consumption across the specifications, there might be a change in the intensity of the GHG emissions (or energy consumption). One possible explanation for why we may see the positive coefficients above is that firms grow, thereby increasing their output and emissions. To test this hypothesis, we repeat the estimation of Eq (1) on the matched sample using proxies for the intensity of the GHG emissions–specifically, as the outcomes we use GHG emissions and energy consumption, both divided by the firm’s sales as a proxy, which allows us to see the change in the GHG emissions and energy consumption per unit revenue. Because we do not have data on the firms’ actual output, the implicit assumption is that commodity prices remain constant throughout the study period, so that all changes in the revenue are driven by output. We then estimate Eq (1) with pre-processed data using the intensity of the GHG emissions and energy consumption as dependent variables.

The negative and statistically significant coefficients on both the intensity of the GHG emissions and the energy consumption indicate that despite the increase in total emissions, firms appear to become more efficient in their production (Table 7). That is, increases in the sales, our proxy for output, outpace increases in the energy consumption and GHG. However, the statistical effectiveness of the effect disappears when we consider synthetic difference-in-difference models (Table 6). This is due to the heterogeneous impact of KETS on the different firms.

**Table 7:** Post-matching difference-in-differences estimates using intensity of GHG/energy.

	(1)	(2)
	Ln(Intensity of GHG)	Ln(Intensity of Energy)
T	0.0523	0.0600
	(0.0581)	(0.0572)
T X D	-0.136*	-0.162**
	(0.0775)	(0.0798)
Observations	346	346
Num. Firms	49	49
FE	Yes	Yes

Standard errors in parentheses Note: repeated observations are considered via frequency weights.

* *p* < .1

** *p* < .05

*** *p* < .01

## Discussion and conclusions

Using rigorous impact evaluation methods, we examine the impact of the first phase of the KETS on GHG emissions and energy consumption by public firms relative to another policy, the TMS. Across multiple specifications, we find higher GHG emissions and energy consumption under the KETS relative to the TMS. These are likely driven by the largest emitters. However, this correlates with increasing sales (our proxy for output) and improved efficiency in the production of the treated firms. In other words, we find that even though total emissions seem to have increased under KETS, production efficiency may have increased. Therefore, we cannot claim that the first phase of the KETS failed to offer incentives for firms to exceed the compliance target.

Our findings are subject to some caveats. First, because of data constraints, we focus on public firms, which may be more responsive to demand shocks and more efficient and may grow faster relative to private firms [27]. However, our results are consistent with previous studies documenting some improvements in efficiency for some industries [24, 25]. Second, apart from small sample size considerations, the large standard errors often render our estimates insignificant; the large standard errors are likely driven by heterogeneity in firm performance. This could be due to multiple factors: First, firm size, specifically when it became targeted by KETS, seems important. Second, that could be due to some firms’ lack of policy awareness and policy uncertainty, as commonly found in the early phase of the EU ETS and China’s ETS pilots: These factors might hinder investment in abatement technology [37–40]. For example, firms under the KETS may perceive the emission allowance not as a tradable asset but as a regulation to comply with, which may deter firms’ market participation and constrains the managerial flexibility of the ETS [41]. Also, firms may adopt a conservative approach (i.e., unwilling to sell excess permits and banking them for future use) in the KETS market participation due to uncertainty over the future policy [41]. Auctioning emission permits and introducing a price ceiling and floor are suggested to increase a firm’s market participation and reduce policy uncertainty [39, 40].

While firms targeted by the KETS can offset their emissions using projects elsewhere, our dataset does not contain information on purchased offsets. However, the total level of emissions for the first phase being below the policy threshold, coupled with the low level of noncompliance (1 firm in 2015 and 2 firms in 2017) [29], suggest that firms across the targeted industries met the policy goals in aggregate. Since our analysis indicates increasing emissions and energy efficiency, firms were likely to invest in purchasing permits (especially in the later years) and use banked permits and offsets to meet the policy targets [42]. For example, in aggregate, 56% of all traded volume during the first phase of KETS was comprised of allowances, with 35% and 9% containing offsets and credits, respectively [42].

Finally, our data do not explicitly allow us to differentiate firm growth. Given that the current KETS design enables the adjustment of allowances when a firm expands (e.g., by installing a new facility), the aggregate caps may need to be increased if multiple firms expand. In the following KETS phases, policymakers need to discuss how to allocate the allowance to growing firms, particularly when the number of growing firms is significant.

### Policy implications

With climate change becoming more and more of a threat to life on the planet, it is important that we curb industrial greenhouse gas emissions. To this end, we need to understand and evaluate the impact of interventions like the KETS. Our findings suggest that KETS can improve overall performance, but there is significant variability in the impacts. Our small sample size precludes us from examining the heterogeneity further (e.g., in terms of spatial patterns or by industry). Therefore, further investigation is necessary to understand and improve the effectiveness of the KETS on emissions.

Some promising avenues for future study include an investigation of the subsequent phases of the KETS. For example, with the introduction of permit auctioning, a tightened cap in the second phase, and increased trading of permits, we might see different firms’ behavior in terms of GHG emissions and energy consumption. Nevertheless, the subsequent phases of the KETS need to limit offsets, given recent concerns about the additionality of carbon credits from offset schemes [43–45]. Other important questions pertain to explicit comparisons of publicly traded and private firms, tests whether technological spillover from large to small firms or from early entrants to latecomers to the regulations exist, analyses of the impact of climate policy on market entry to new markets, and demand for the greener products. In addition, we need to know whether the benefits under each policy exceed private and public costs. Our work is a first step in understanding the impact of KETS and the mechanisms underpinning its impact.

## Supporting information

S1 Table Checking trends between treatment and control groups. (DOCX)

S2 Table Mean comparisons before and after matching. (DOCX)

S1 Fig Propensity score distribution by treatment status. (TIF)

S2 Fig Synthetic difference-in-differences weights for groups 1 and 13 in ln(GHG). (TIF)

S3 Fig Synthetic difference-in-differences trends for groups 1 and 13 in ln(GHG). (TIF)

S4 Fig Synthetic difference-in-differences weights for groups 2 and 14 in ln(GHG). (TIF)

S5 Fig Synthetic difference-in-differences trends for groups 2 and 14 in ln(GHG). (TIF)

S6 Fig Synthetic difference-in-differences weights for groups 3 and 15 in ln(GHG). (TIF)

S7 Fig Synthetic difference-in-differences for groups 3 and 15 in ln(GHG). (TIF)

S8 Fig Synthetic difference-in-differences weights for groups 1,2,3 and 13,14,15in ln(GHG). (TIF)

S9 Fig Synthetic difference-in-differences trends for groups 1,2,3 and 13,14,15 in ln(GHG). (TIF)

S10 Fig Synthetic difference-in-differences weights for groups 1 and 13 in ln(energy). (TIF)

S11 Fig Synthetic difference-in-differences trends for groups 1and 13 in ln(energy). (TIF)

S12 Fig Synthetic difference-in-differences weights for groups 2 and 14 in ln(energy). (TIF)

S13 Fig Synthetic difference-in-differences trends for groups 2 and 14 in ln(energy). (TIF)

S14 Fig Synthetic difference-in-differences weights for groups 3 and 15 in ln(energy). (TIF)

S15 Fig Synthetic difference-in-differences trends for groups 3 and 15 in ln(energy). (TIF)

S16 Fig Synthetic difference-in-differences weights for groups 1,2,3 and 13,14,15 in ln(energy). (TIF)

S17 Fig Synthetic difference-in-differences trends for groups 1,2,3 and 13,14,15 in ln(energy). (TIF)

S18 Fig Synthetic difference-in-differences weights for groups 1 and 13 in ln(intensity of GHG). (TIF)

S19 Fig Synthetic difference-in-differences trends for groups 1 and 13 in ln(intensity of GHG). (TIF)

S20 Fig Synthetic difference-in-differences weights for groups 2 and 14 in ln(intensity of GHG). (TIF)

S21 Fig Synthetic difference-in-differences trends for groups 2 and 14 in ln(intensity of GHG). (TIF)

S22 Fig Synthetic difference-in-differences weights for groups 3 and 15 in ln(intensity of GHG). (TIF)

S23 Fig Synthetic difference-in-differences trends for groups 3 and 15 in ln(intensity of GHG). (TIF)

S24 Fig Synthetic difference-in-differences weights for groups 1,2,3 and 13,14,15 in ln(intensity of GHG). (TIF)

S25 Fig Synthetic difference-in-differences trends for groups 1,2,3 and 13,14,15 in ln(intensity of GHG). (TIF)

S26 Fig Synthetic difference-in-differences weights for groups 1 and 13 in ln(intensity of energy). (TIF)

S27 Fig Synthetic difference-in-differences trends for groups 1 and 13 in ln(intensity of energy). (TIF)

S28 Fig Synthetic difference-in-differences weights for groups 2 and 14 in ln(intensity of energy). (TIF)

S29 Fig Synthetic difference-in-differences trends for groups 2 and 14 in ln(intensity of energy). (TIF)

S30 Fig Synthetic difference-in-differences weights for groups 3 and 15 in ln(intensity of energy). (TIF)

S31 Fig Synthetic difference-in-differences trends for groups 3 and 15 in ln(intensity of energy). (TIF)

S32 Fig Synthetic difference-in-differences weights for groups 1,2,3 and 13,14,15 in ln(intensity of energy). (TIF)

S33 Fig Synthetic difference-in-differences trends for groups 1,2,3 and 13,14,15 in ln(intensity of energy). (TIF)
