# Correction: Impact of Korea’s emissions trading scheme on publicly traded firms

**DOI:** 10.1371/journal.pone.0306209

**Published:** 2024-06-21

**Authors:** Nyonho Oh, Daniela A. Miteva, Yehchan Lee

The first author’s name is spelled incorrectly. The correct name is: Nyonho Oh. The correct citation is: Oh N, Miteva DA, Lee Y (2023) Impact of Korea’s emissions trading scheme on publicly traded firms. PLoS ONE 18(5): e0285863. https://doi.org/10.1371/journal.pone.0285863.
