# Supplementary material for: Impact of Korea’s emissions trading scheme on publicly traded firms
Source: PLoS One. 2023 May 24;18(5):e0285863. doi: 10.1371/journal.pone.0285863 (PMC10208515; doi:10.1371/journal.pone.0285863)
Supplement: S1 Table — (DOCX) [file pone.0285863.s001.docx]

S1 Table. Check trends between treatment and control groups

|  | (1) | (2) |
| --- | --- | --- |
|  | Ln(GHG) | Ln(Energy) |
| 2011 X Treated | 0.0283 | 0.264 |
|  | (0.747) | (0.590) |
| 2012 X Treated | -0.125 | -0.0582 |
|  | (0.353) | (0.313) |
| 2013 X Treated | -0.254 | -0.213 |
|  | (0.287) | (0.268) |
| 2015 X Treated | 0.00626 | 0.00581 |
|  | (0.207) | (0.194) |
| 2016 X Treated | 0.0284 | 0.0366 |
|  | (0.208) | (0.195) |
| 2017 X Treated | 0.0832 | 0.0782 |
|  | (0.208) | (0.196) |
| Observations | 1314 | 1314 |
| Control | Yes | Yes |
| Year FE | Yes | Yes |
| Num. firms | 205 | 205 |

Standard errors in parentheses

Control includes Ln(Sales), ROA, and debt ratio

^*^ *p* < .1, ^**^ *p* < .05, ^***^ *p* < .01
