# Supplementary material for: Impact of Korea’s emissions trading scheme on publicly traded firms
Source: PLoS One. 2023 May 24;18(5):e0285863. doi: 10.1371/journal.pone.0285863 (PMC10208515; doi:10.1371/journal.pone.0285863)
Supplement: S2 Table — (DOCX) [file pone.0285863.s002.docx]

S2 Table. Mean comparisons before and after matching

|  | Control | Treated | Normalized difference ^a^ |
| --- | --- | --- | --- |
| *A. Before Matching* |  |  |  |
| Average GHG before 2015 | 23,254.33 | 1,271,719.34 | 0.288 |
|  | (16,212.18) | (6,120,416.74) |  |
| Average Energy before 2015 | 462.76 | 15,499.50 | 0.344 |
|  | (330.11) | (61,850.34) |  |
| Average Sales before 2015 | 1,644,619,055.24 | 6,813,791,757.40 | 0.349 |
|  | (5741439416.40) | (20130846573.52) |  |
| Average ROA before 2015 | 2.14 | 2.26 | 0.024 |
|  | (5.98) | (4.68) |  |
| Average Debt ratio before 2015 | 0.49 | 0.51 | 0.133 |
|  | (0.20) | (0.19) |  |
| Num. firms | 37 | 168 |  |
|  |  |  |  |
| *B. After Matching* |  |  |  |
| Average GHG before 2015 | 19,602.53 | 38,600.37 | 0.004 |
|  | (2,390.47) | (13,189.50) |  |
| Average Energy before 2015 | 378.39 | 807.89 | 0.01 |
|  | (50.21) | (526.84) |  |
| Average Sales before 2015 | 845,087,268.00 | 593,913,839.35 | 0.018 |
|  | (858,546,796.16) | (674,842,252.73) |  |
| Average ROA before 2015 | 2.43 | 2.10 | 0.077 |
|  | (3.21) | (3.04) |  |
| Average Debt ratio before 2015 | 0.53 | 0.53 | 0.006 |
|  | (0.16) | (0.16) |  |
| Num. firms | 18 | 31 |  |

Standard deviations in parentheses

the following formula calculates a Normalized difference: $\frac{│\bar{x}_{treat}-\bar{x}_{control}│}{\sqrt{\left( s_{treat}^{2}+s_{control}^{2} \right)/2}}$, where $s^{2}$ indicates standard deviations and $\bar{x}$ the mean for a covariate for the treatment and control groups, respectively.
