# Supplementary figures and images for: Impact of Korea’s emissions trading scheme on publicly traded firms
Source: PLoS One. 2023 May 24;18(5):e0285863. doi: 10.1371/journal.pone.0285863 (PMC10208515; doi:10.1371/journal.pone.0285863)

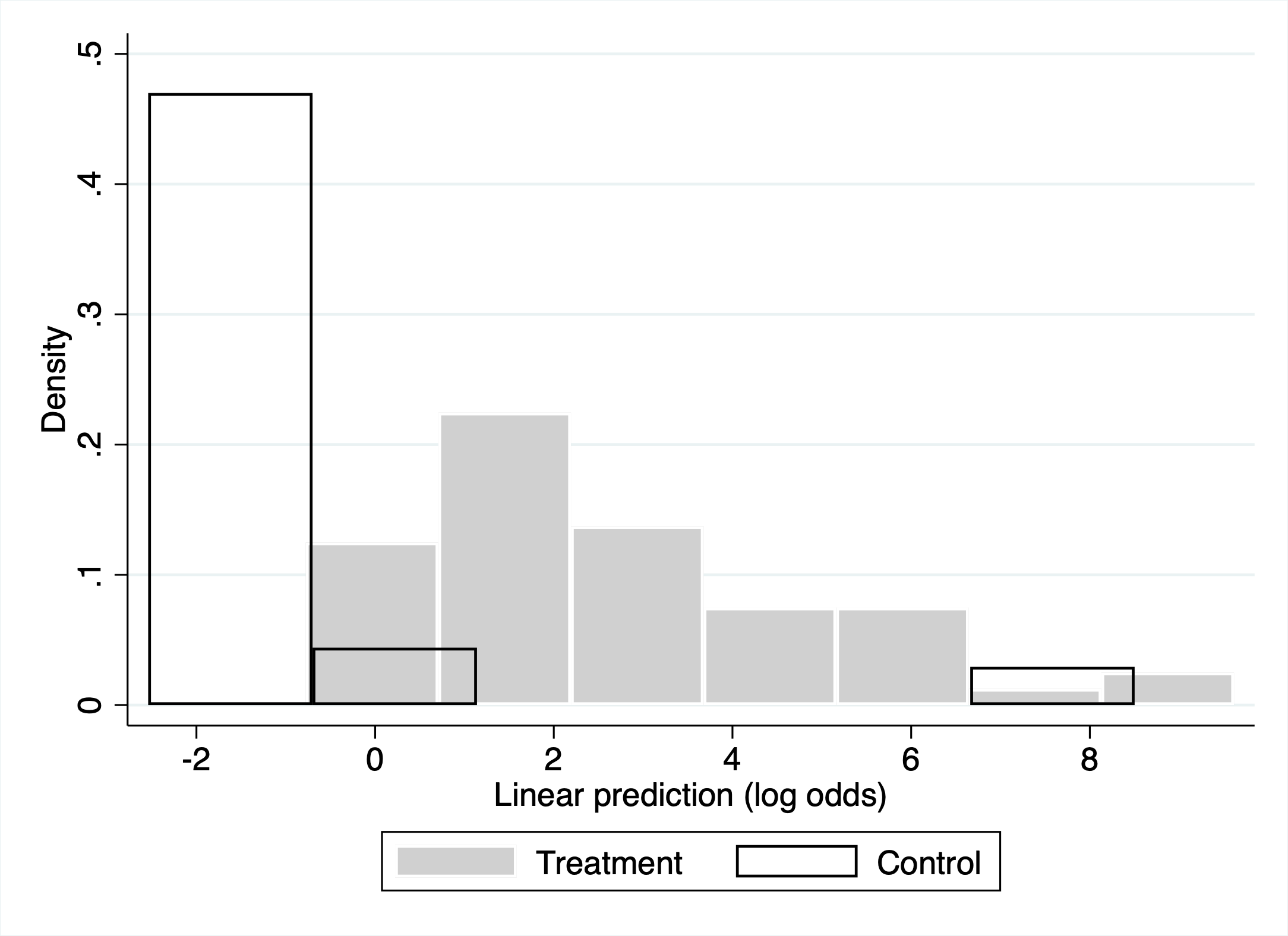

Supplement: S1 Fig — (TIF) [file pone.0285863.s003.tif]

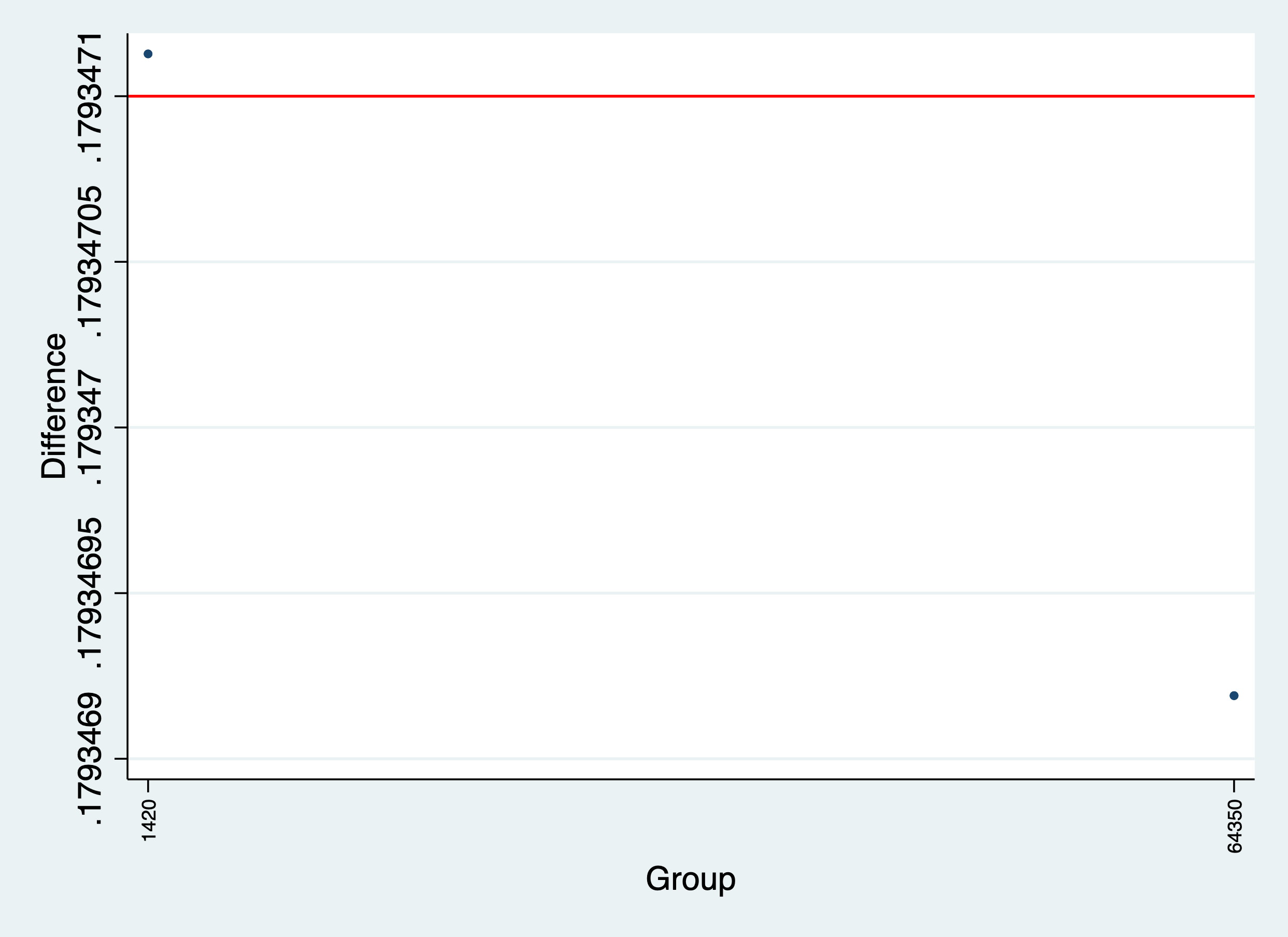

Supplement: S2 Fig — (TIF) [file pone.0285863.s004.tif]

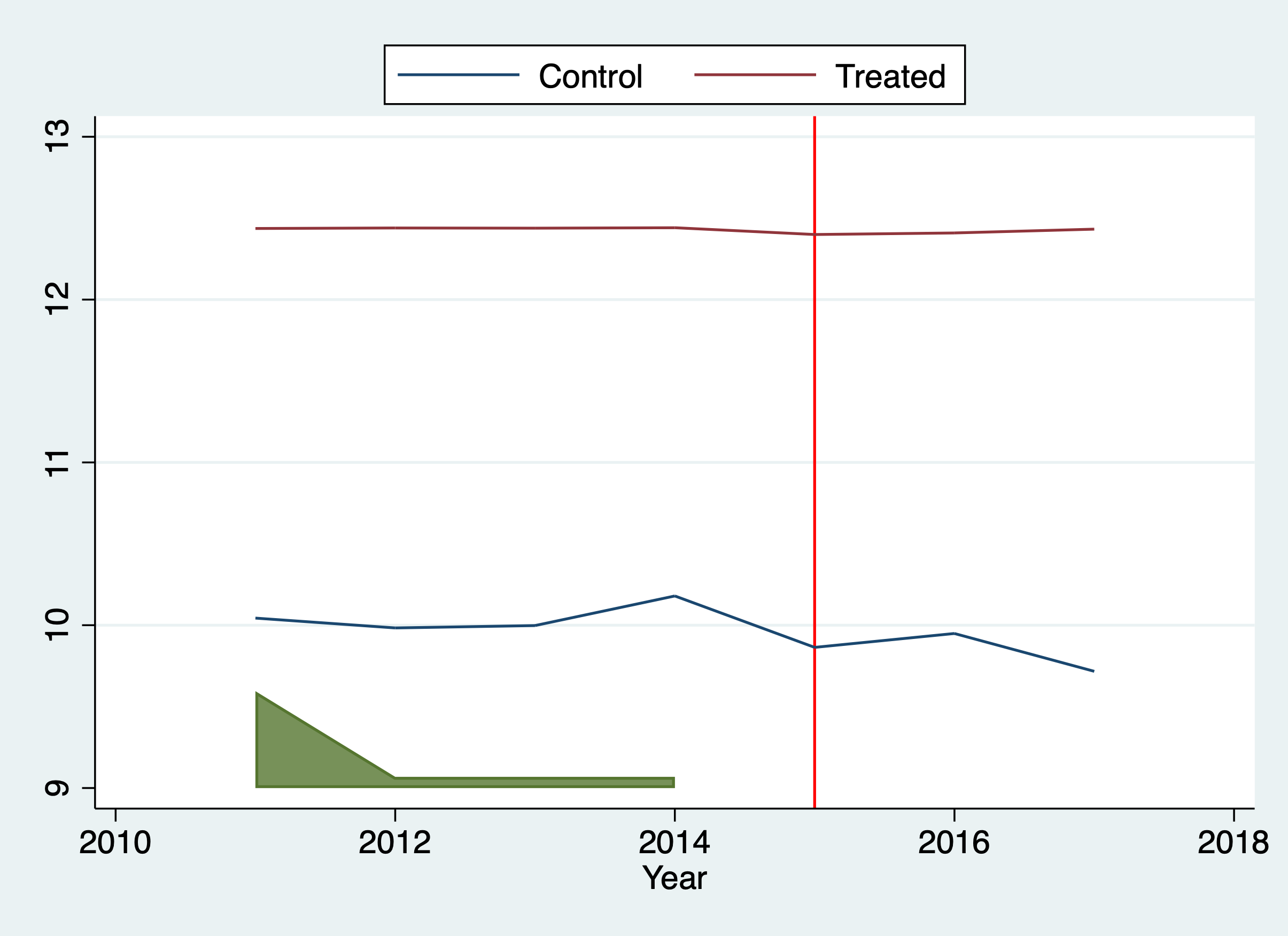

Supplement: S3 Fig — (TIF) [file pone.0285863.s005.tif]

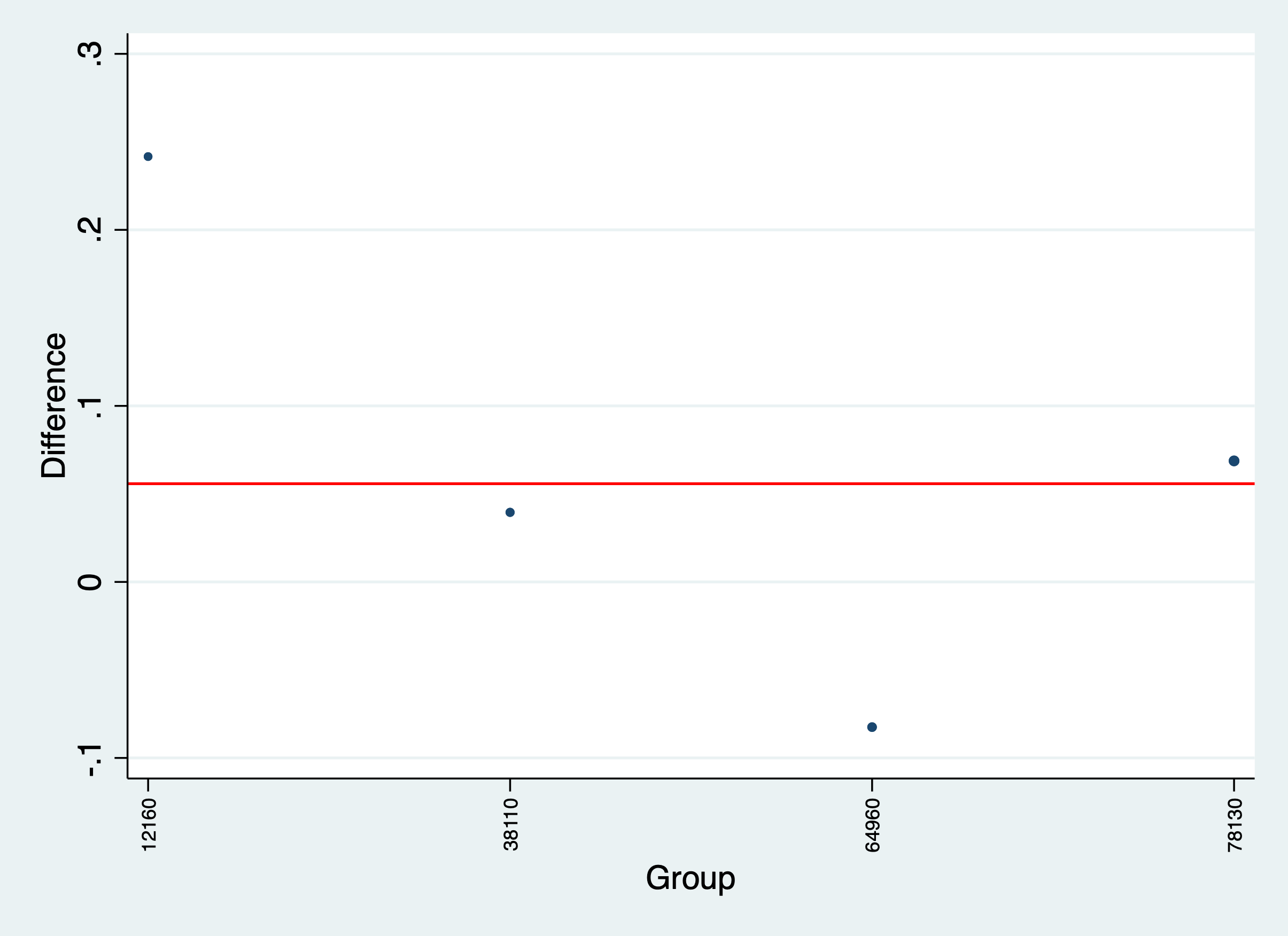

Supplement: S4 Fig — (TIF) [file pone.0285863.s006.tif]

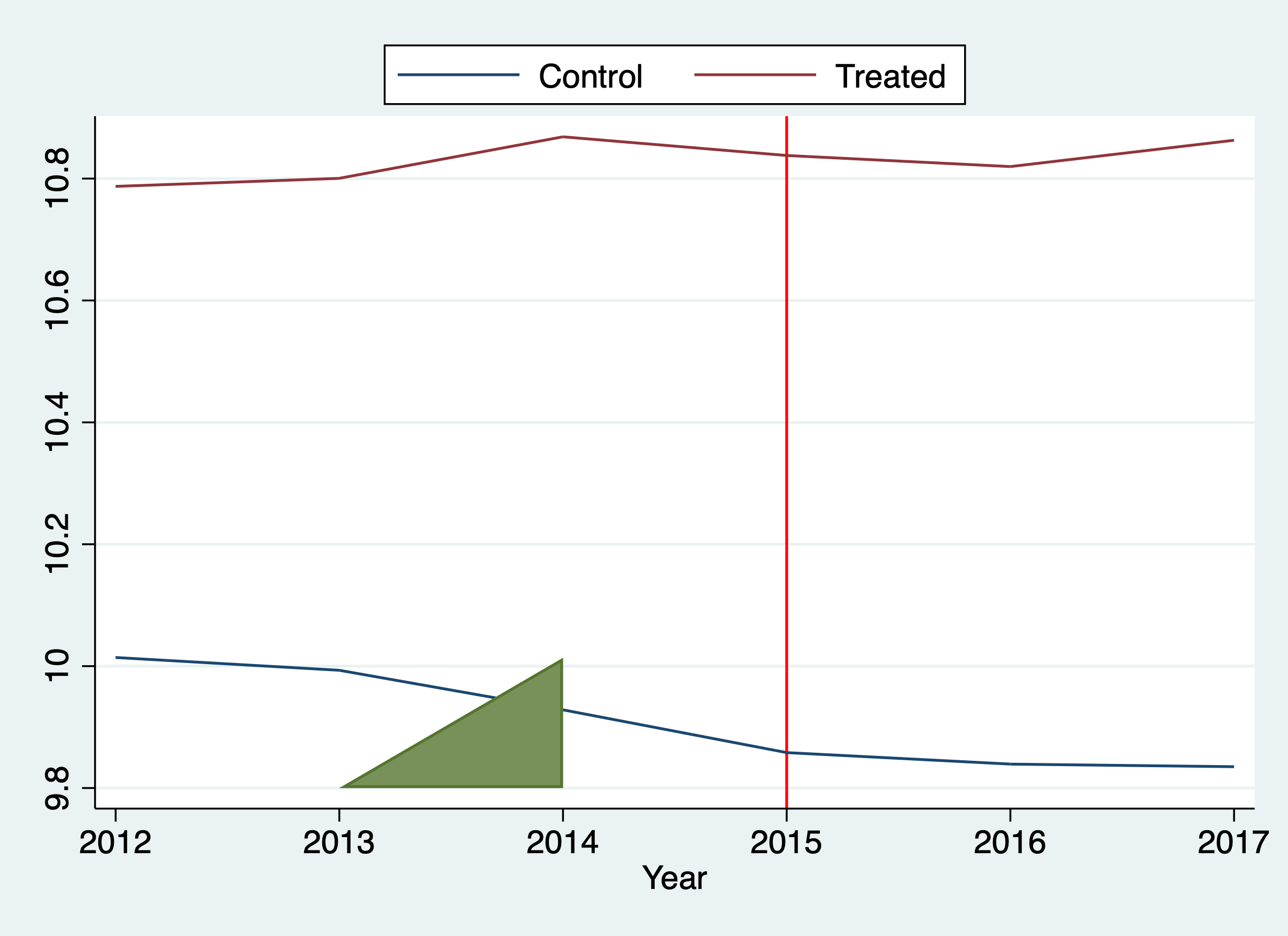

Supplement: S5 Fig — (TIF) [file pone.0285863.s007.tif]

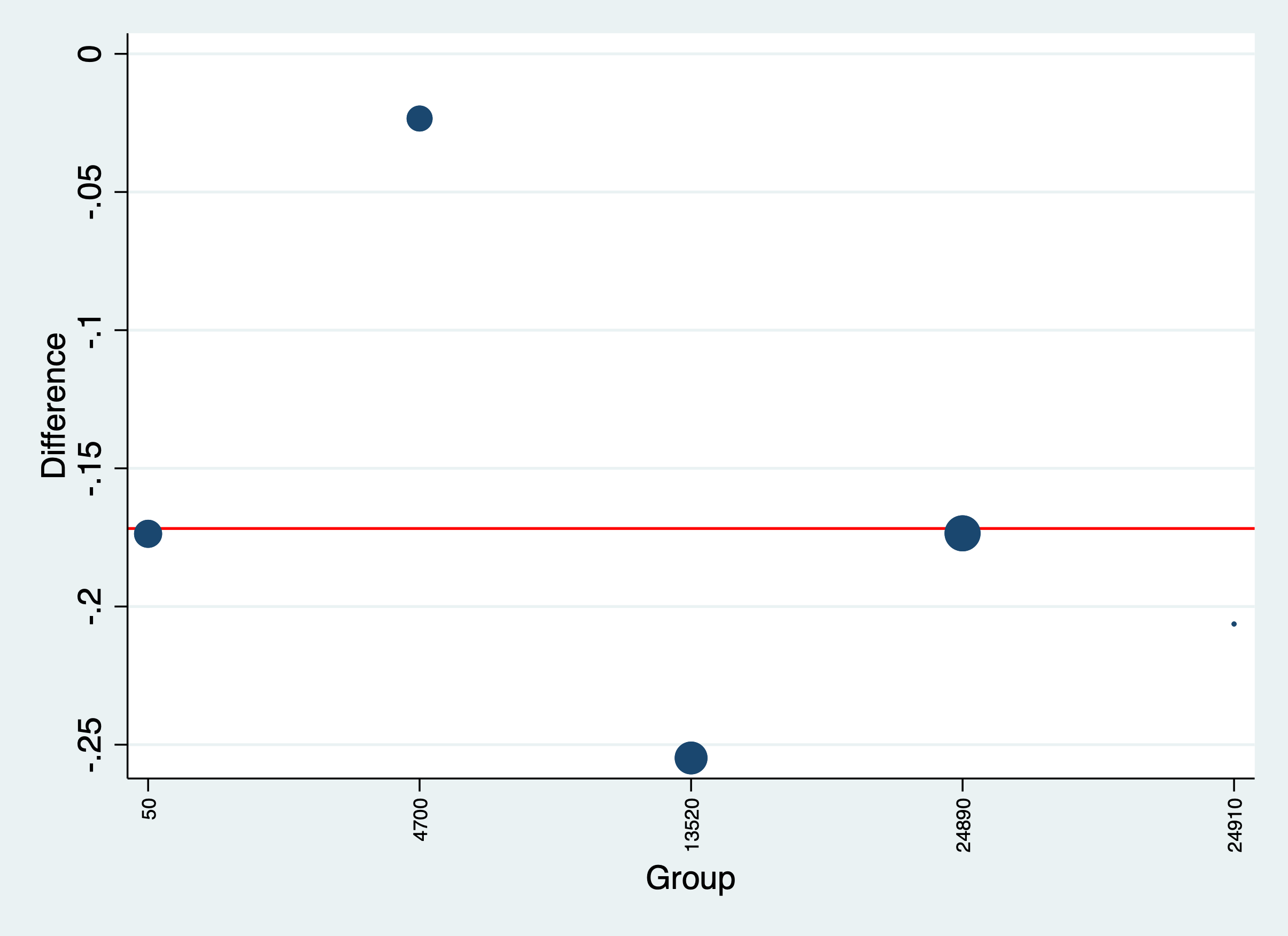

Supplement: S6 Fig — (TIF) [file pone.0285863.s008.tif]

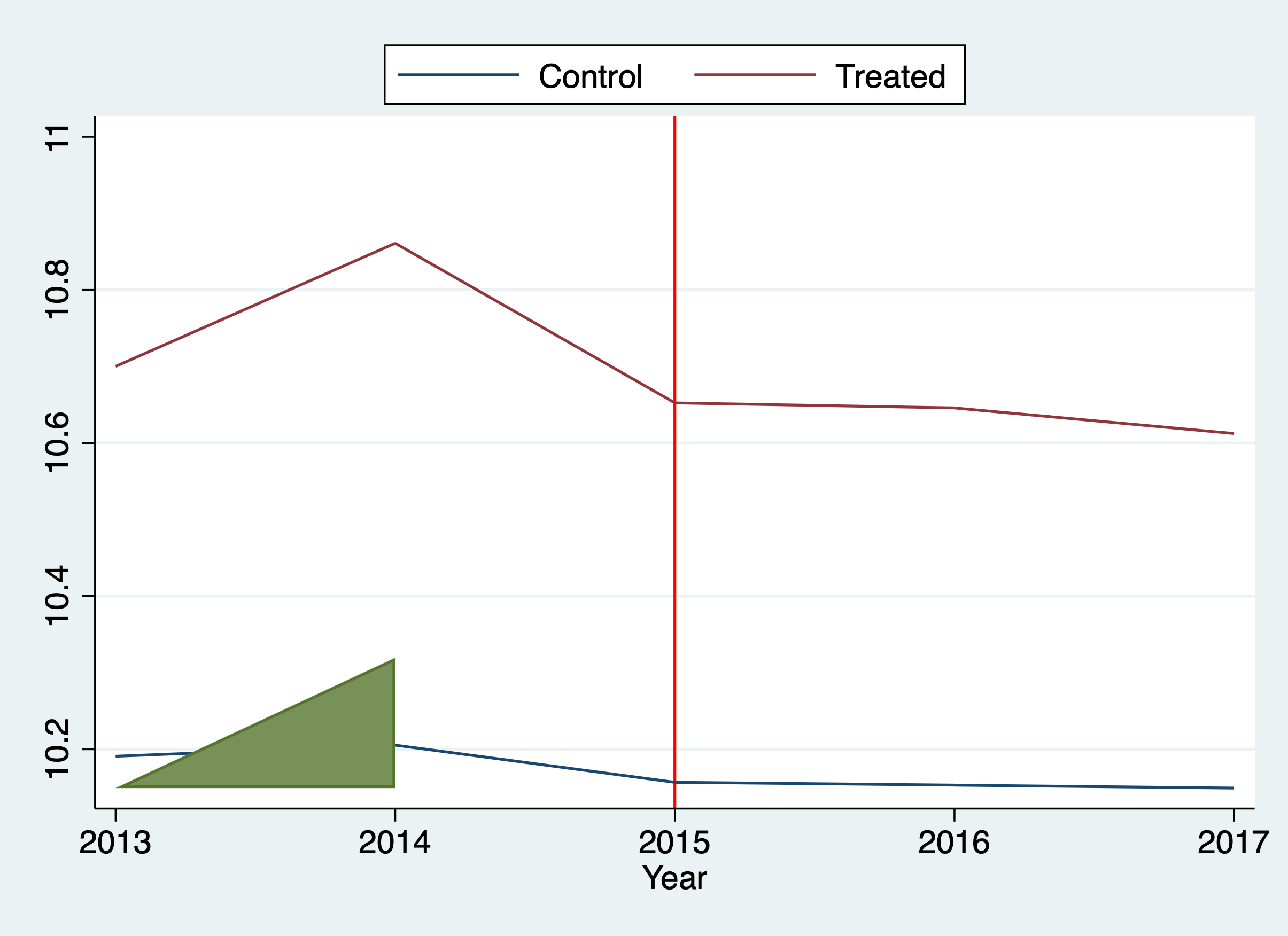

Supplement: S7 Fig — (TIF) [file pone.0285863.s009.tif]

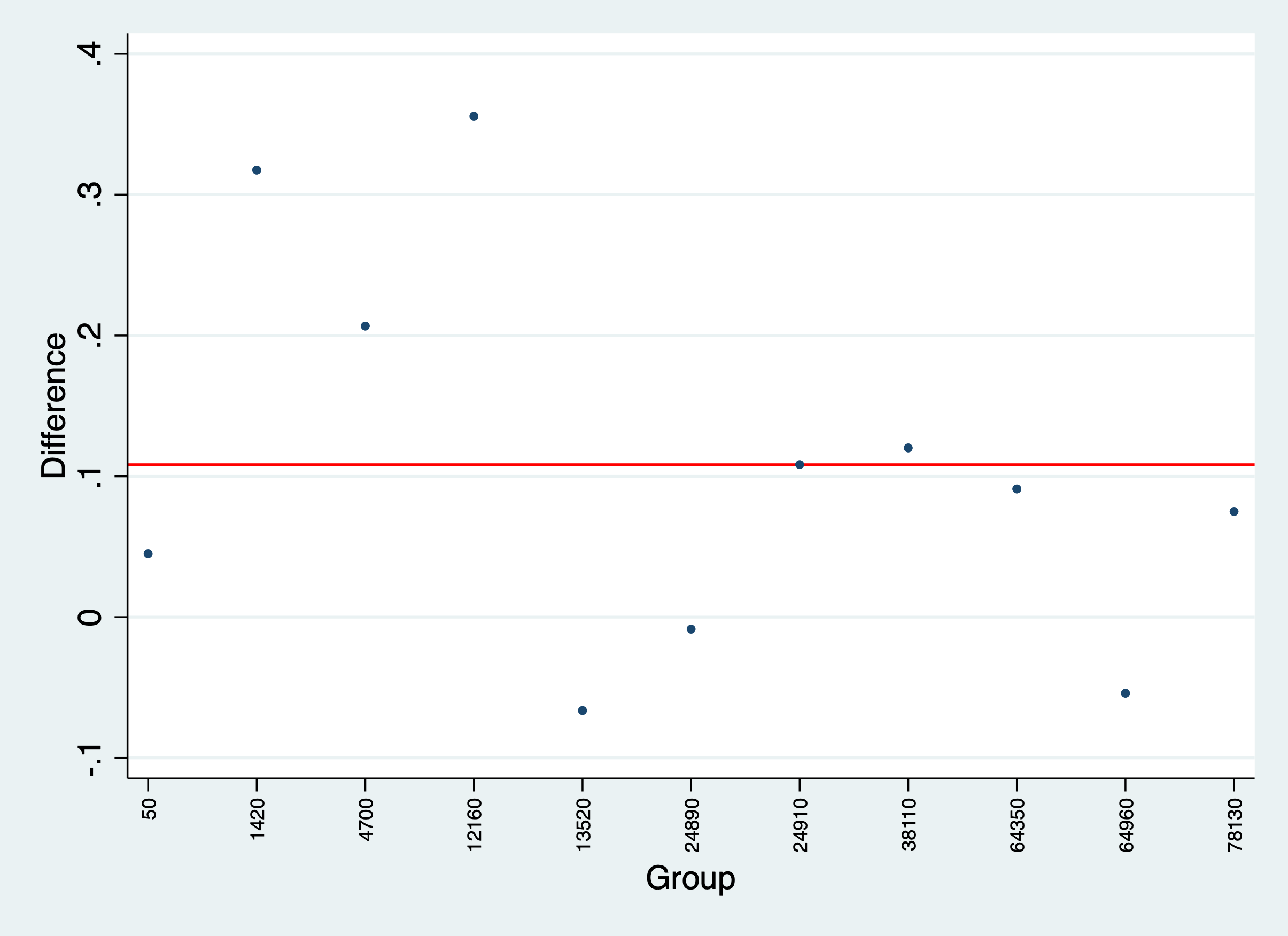

Supplement: S8 Fig — (TIF) [file pone.0285863.s010.tif]

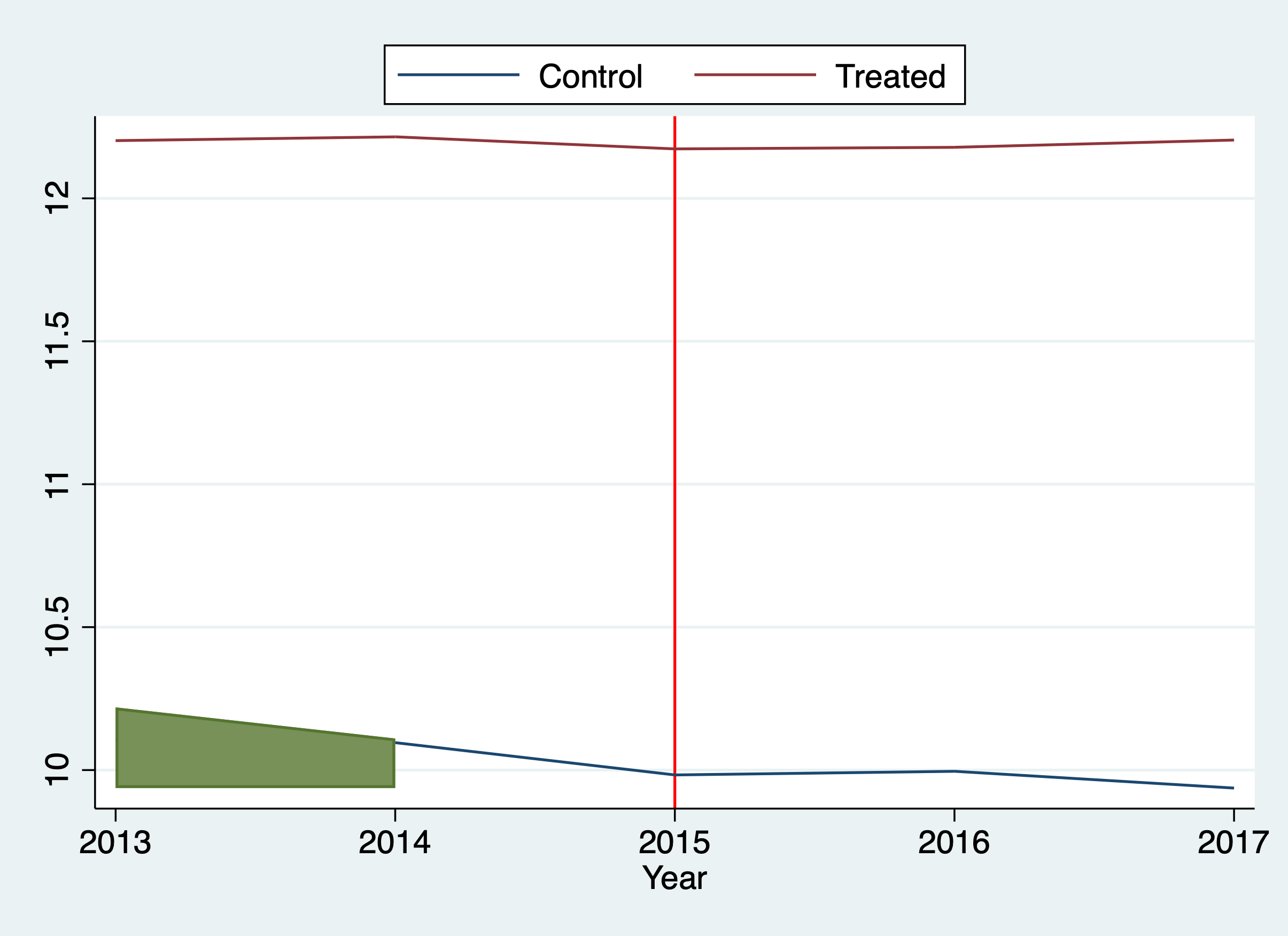

Supplement: S9 Fig — (TIF) [file pone.0285863.s011.tif]

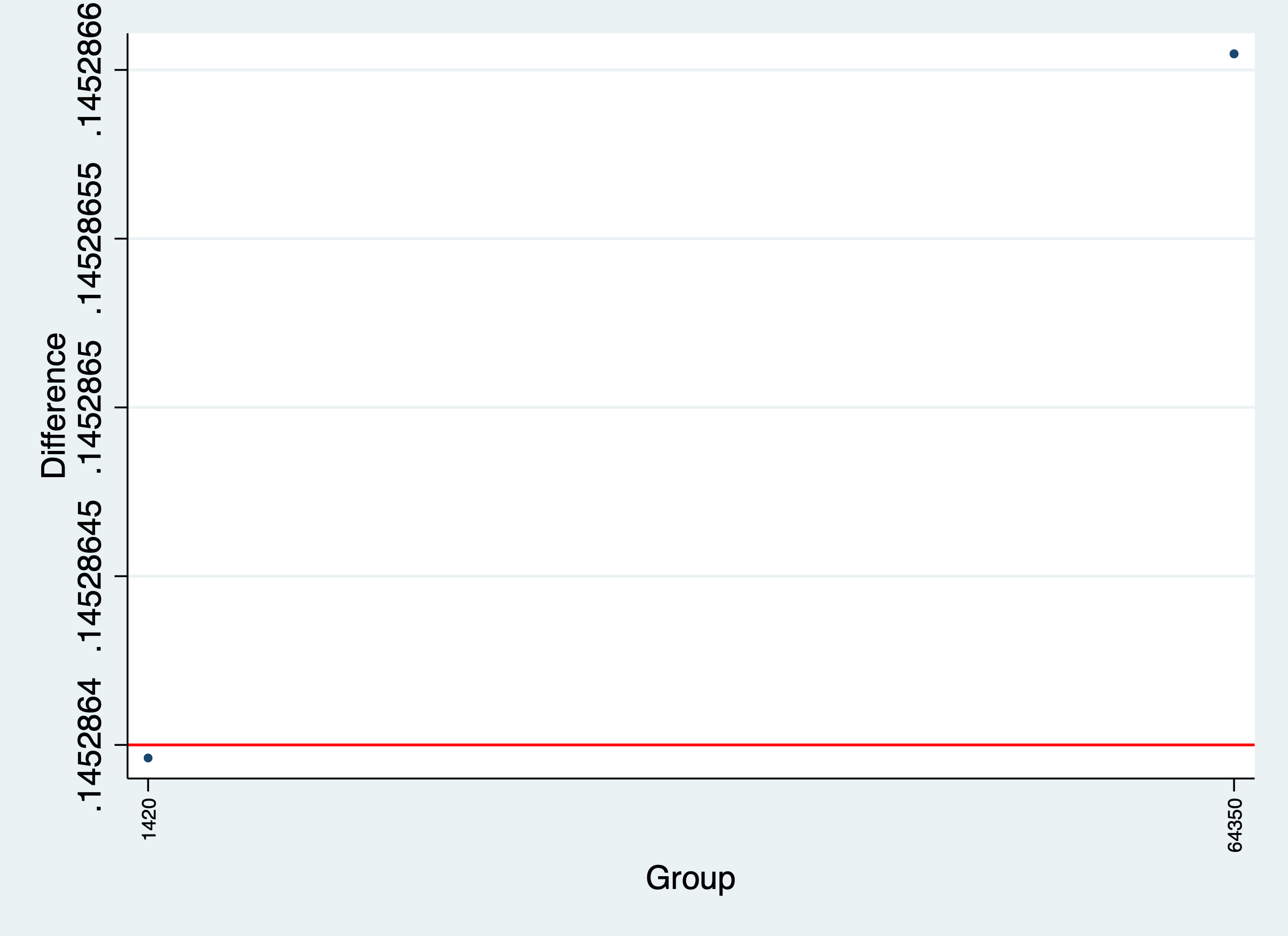

Supplement: S10 Fig — (TIF) [file pone.0285863.s012.tif]

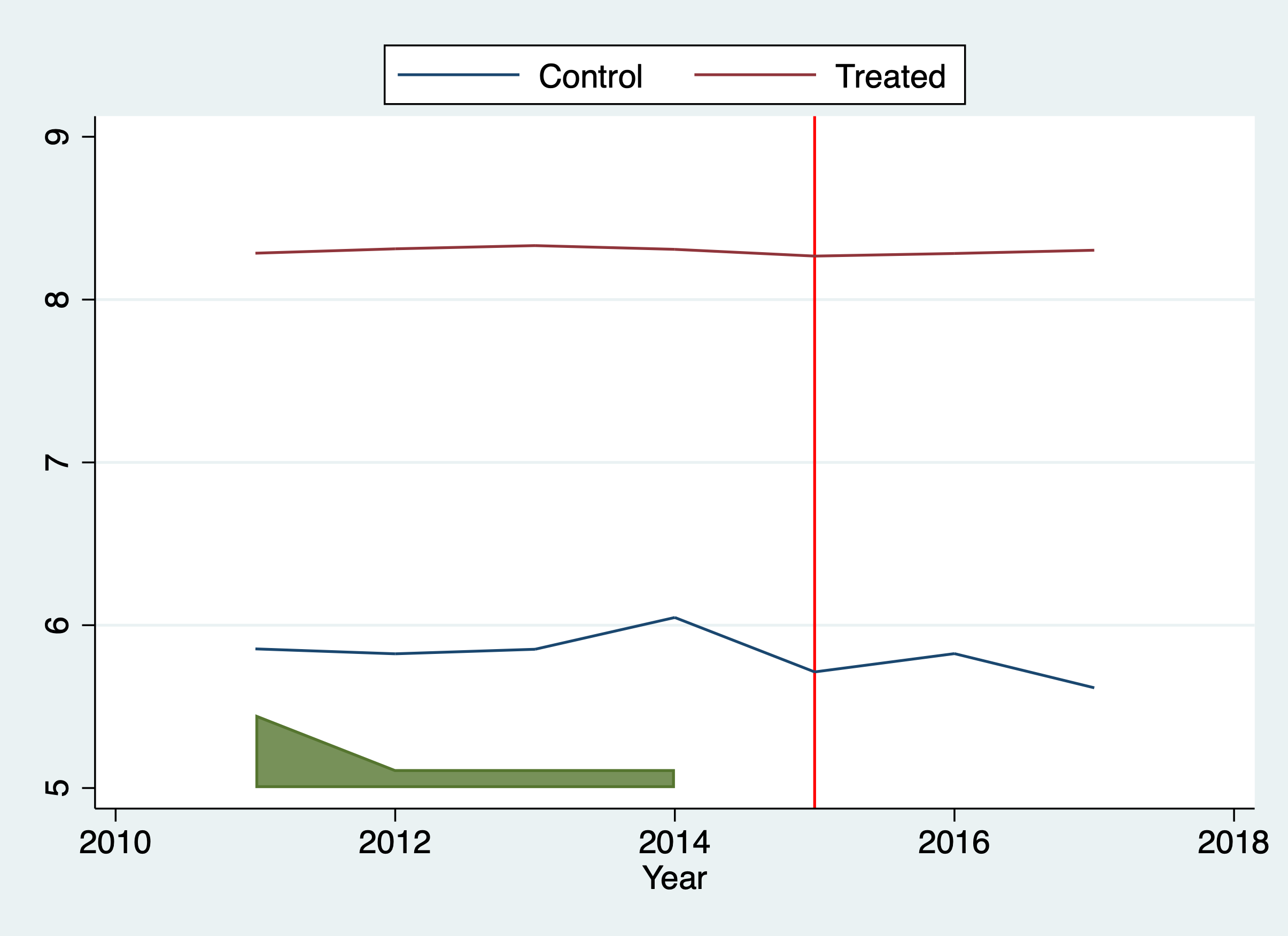

Supplement: S11 Fig — (TIF) [file pone.0285863.s013.tif]

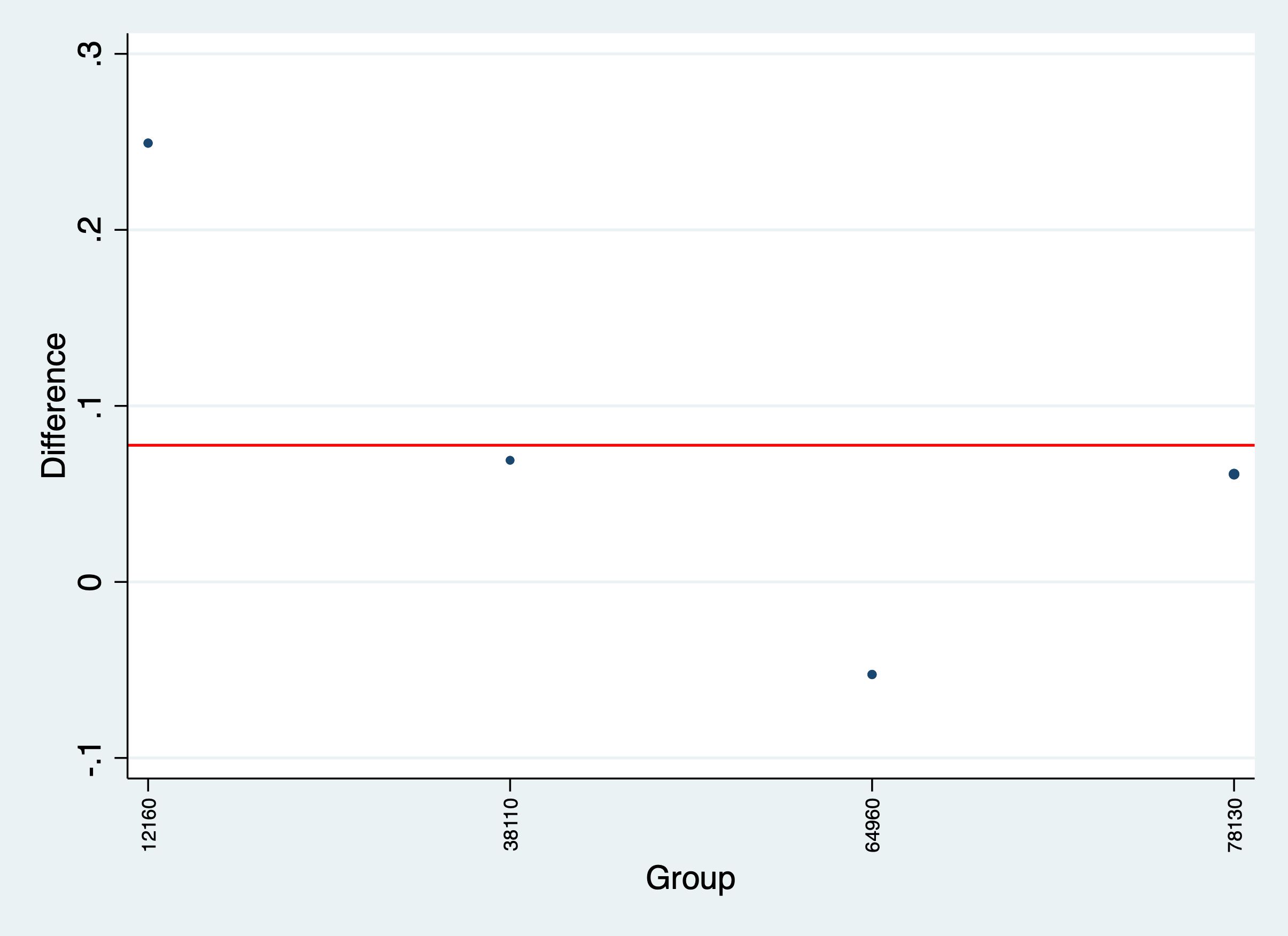

Supplement: S12 Fig — (TIF) [file pone.0285863.s014.tif]

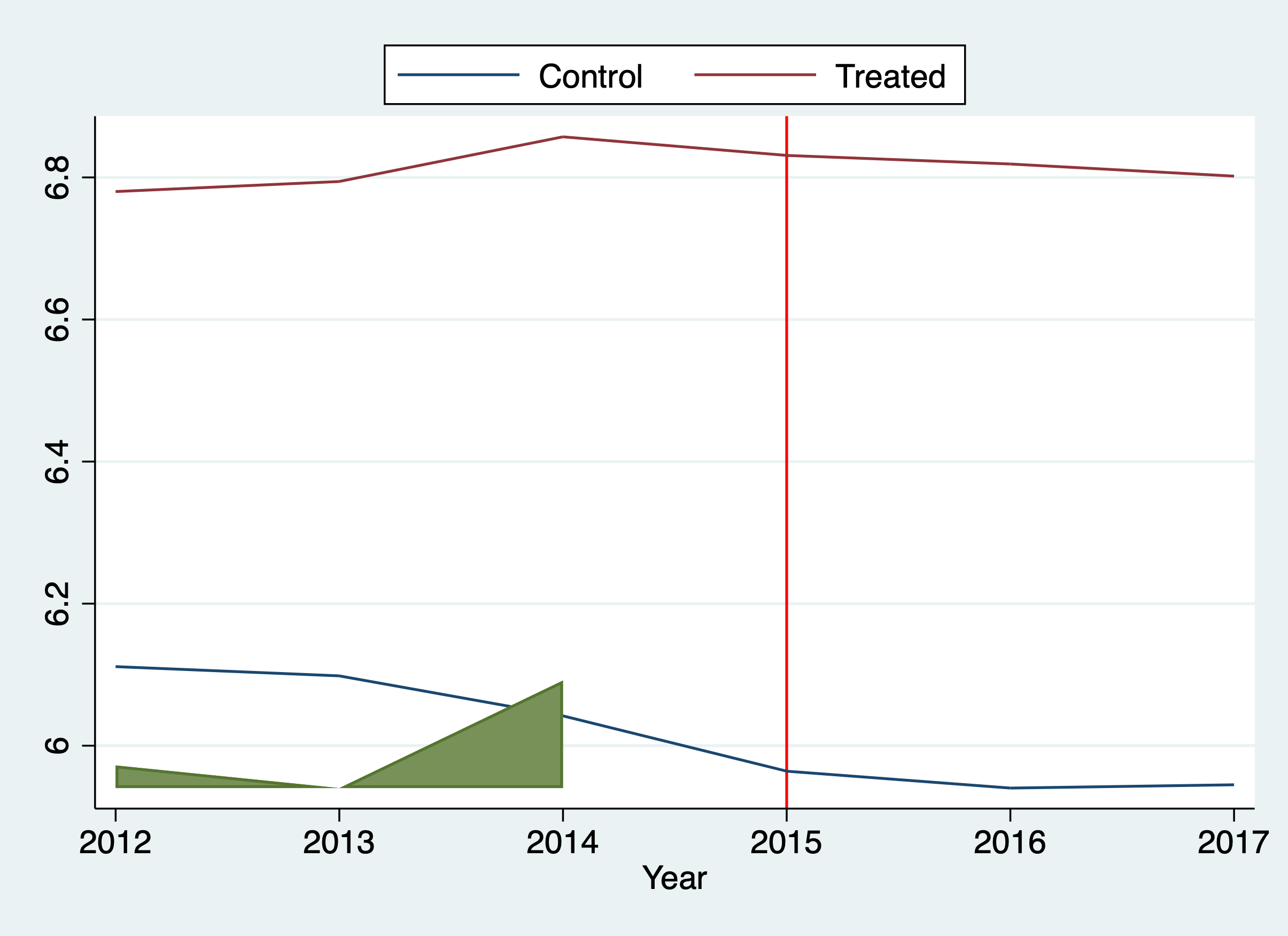

Supplement: S13 Fig — (TIF) [file pone.0285863.s015.tif]

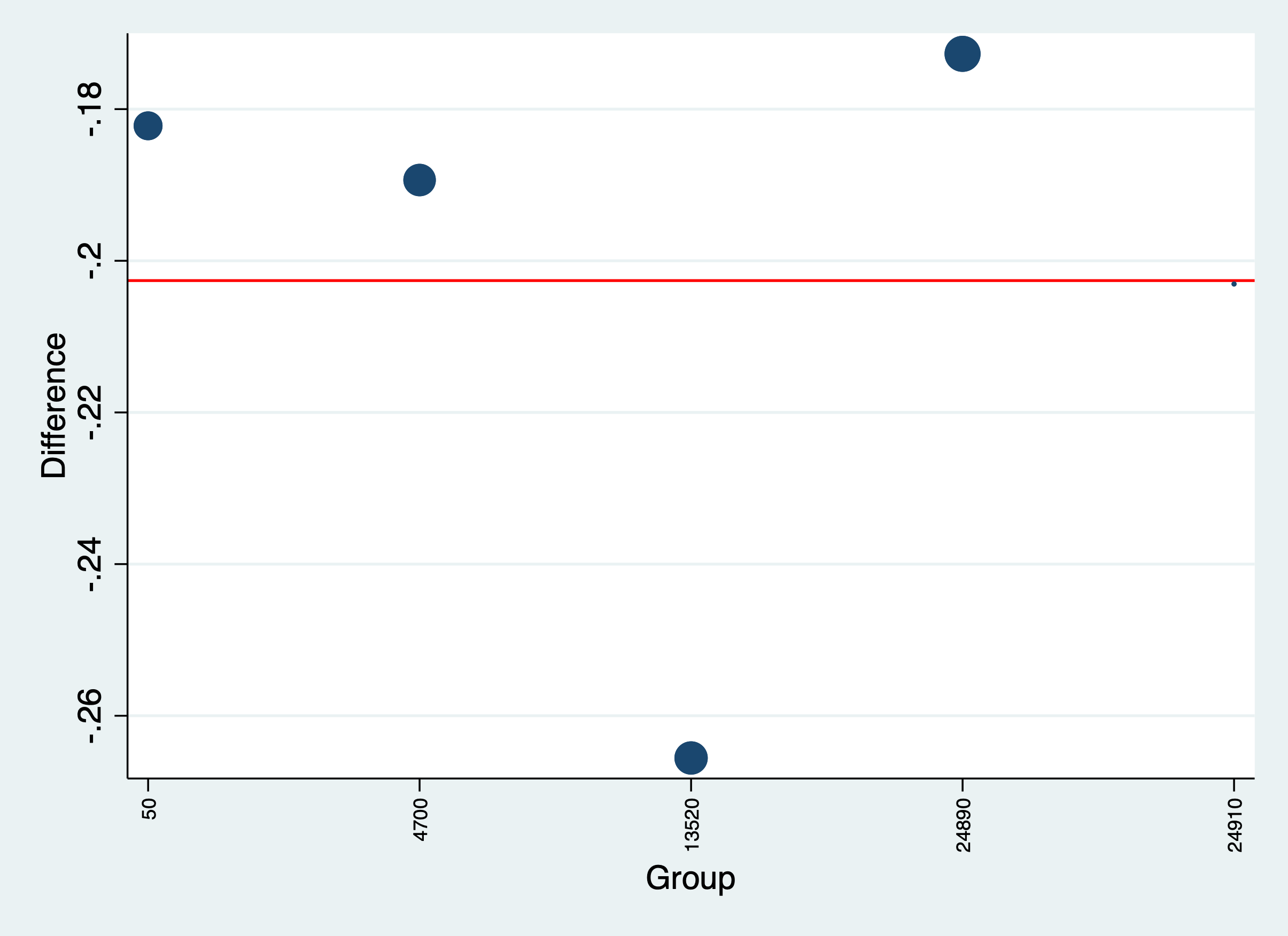

Supplement: S14 Fig — (TIF) [file pone.0285863.s016.tif]

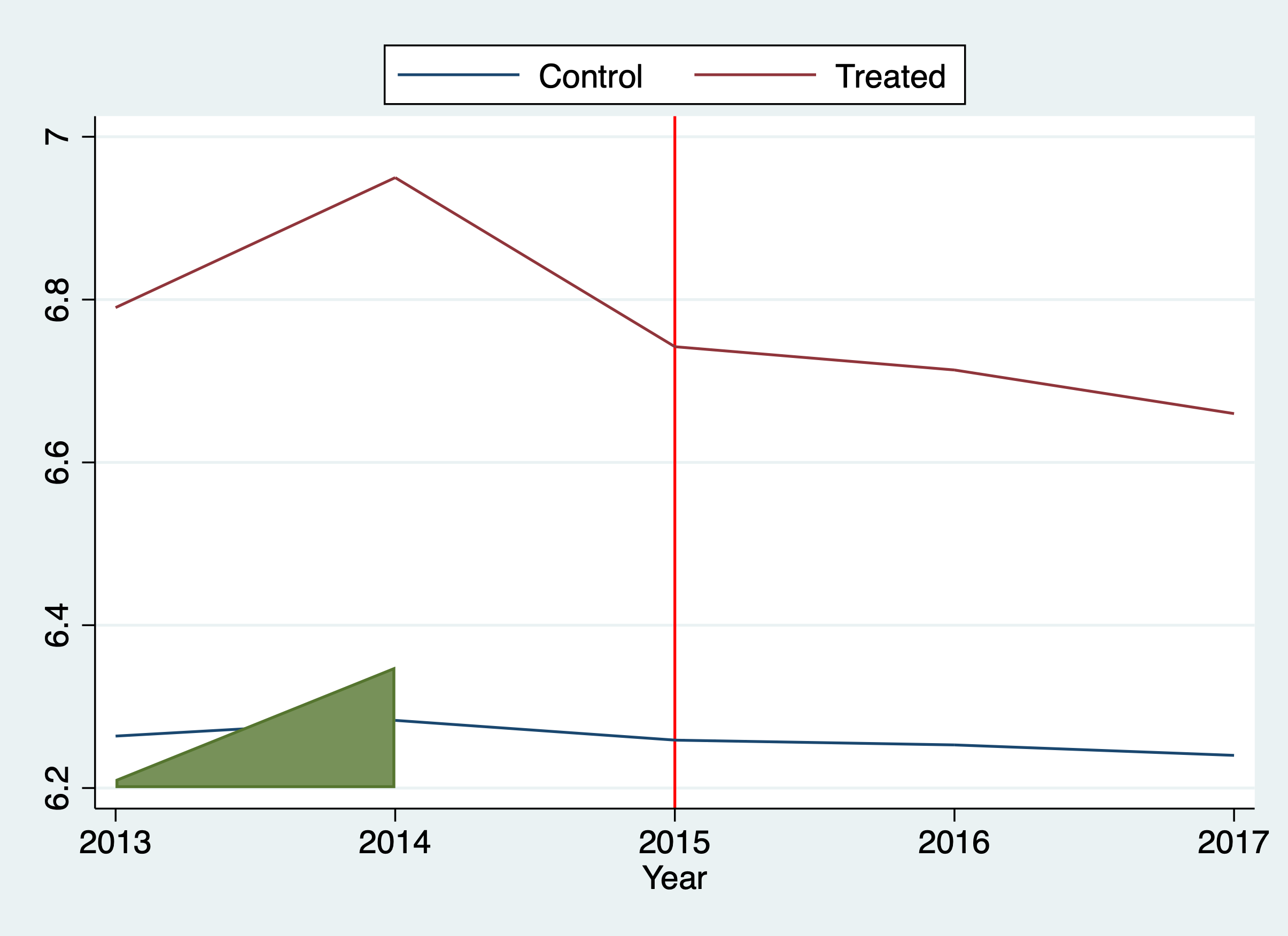

Supplement: S15 Fig — (TIF) [file pone.0285863.s017.tif]

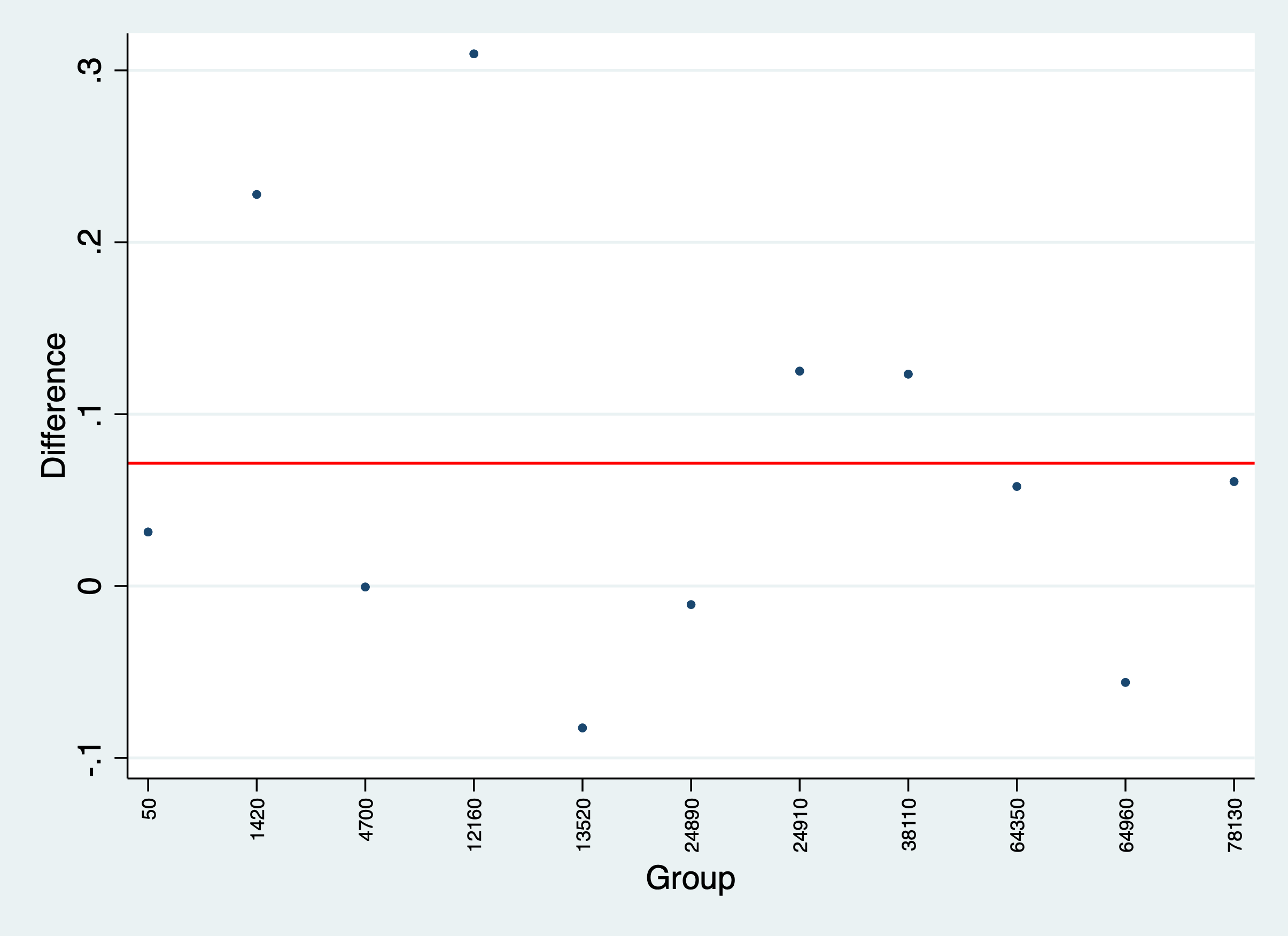

Supplement: S16 Fig — (TIF) [file pone.0285863.s018.tif]

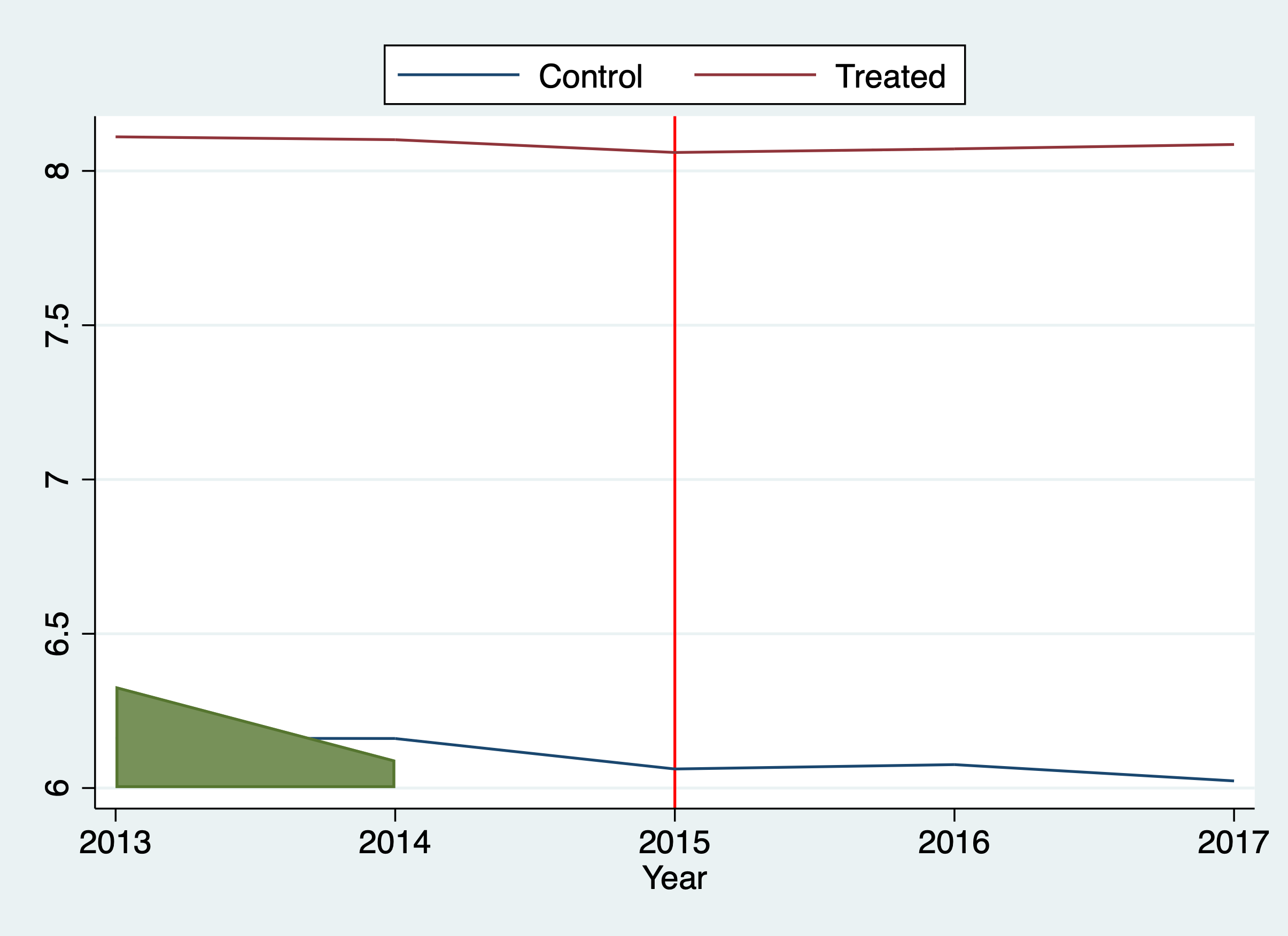

Supplement: S17 Fig — (TIF) [file pone.0285863.s019.tif]

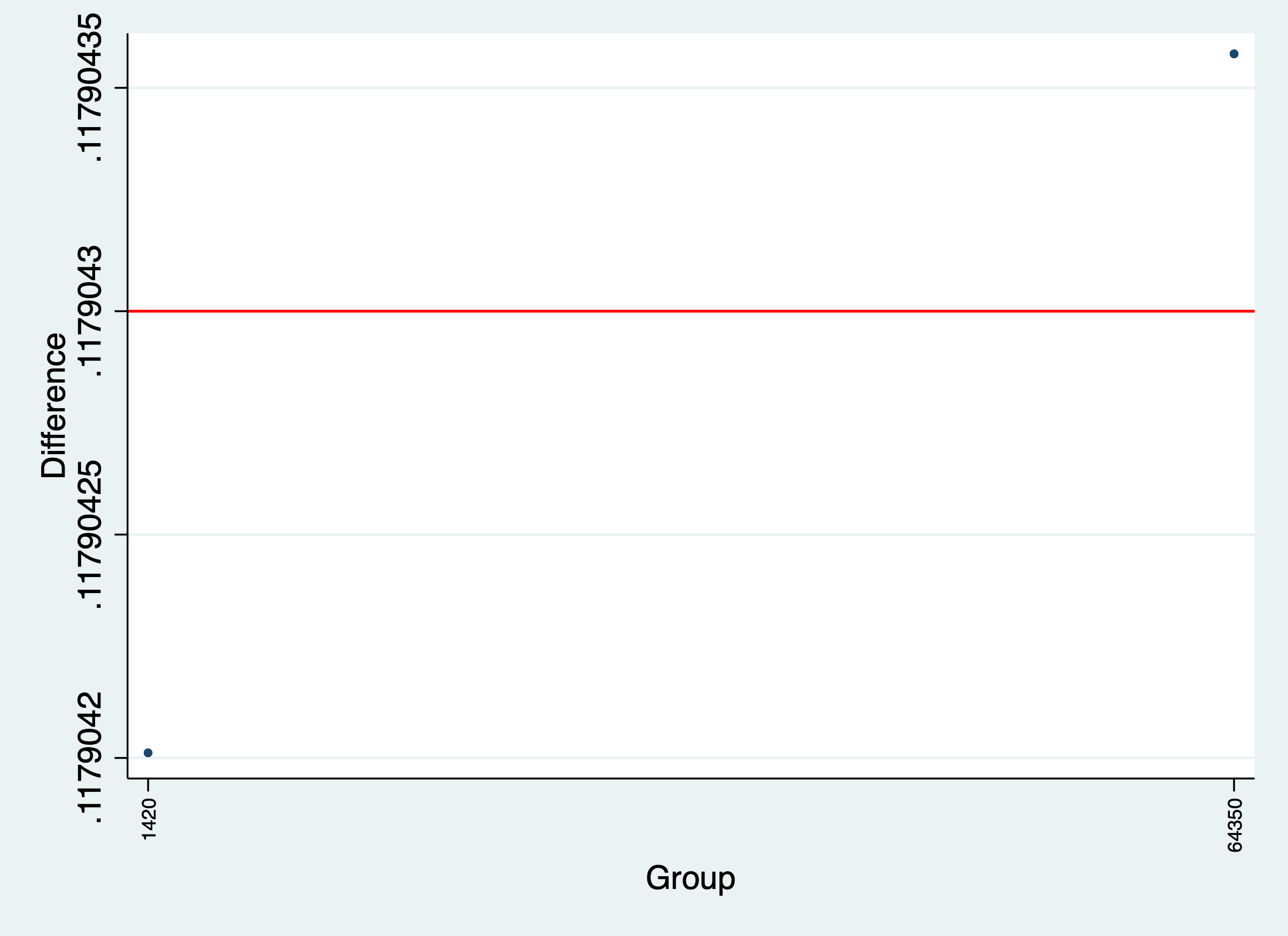

Supplement: S18 Fig — (TIF) [file pone.0285863.s020.tif]

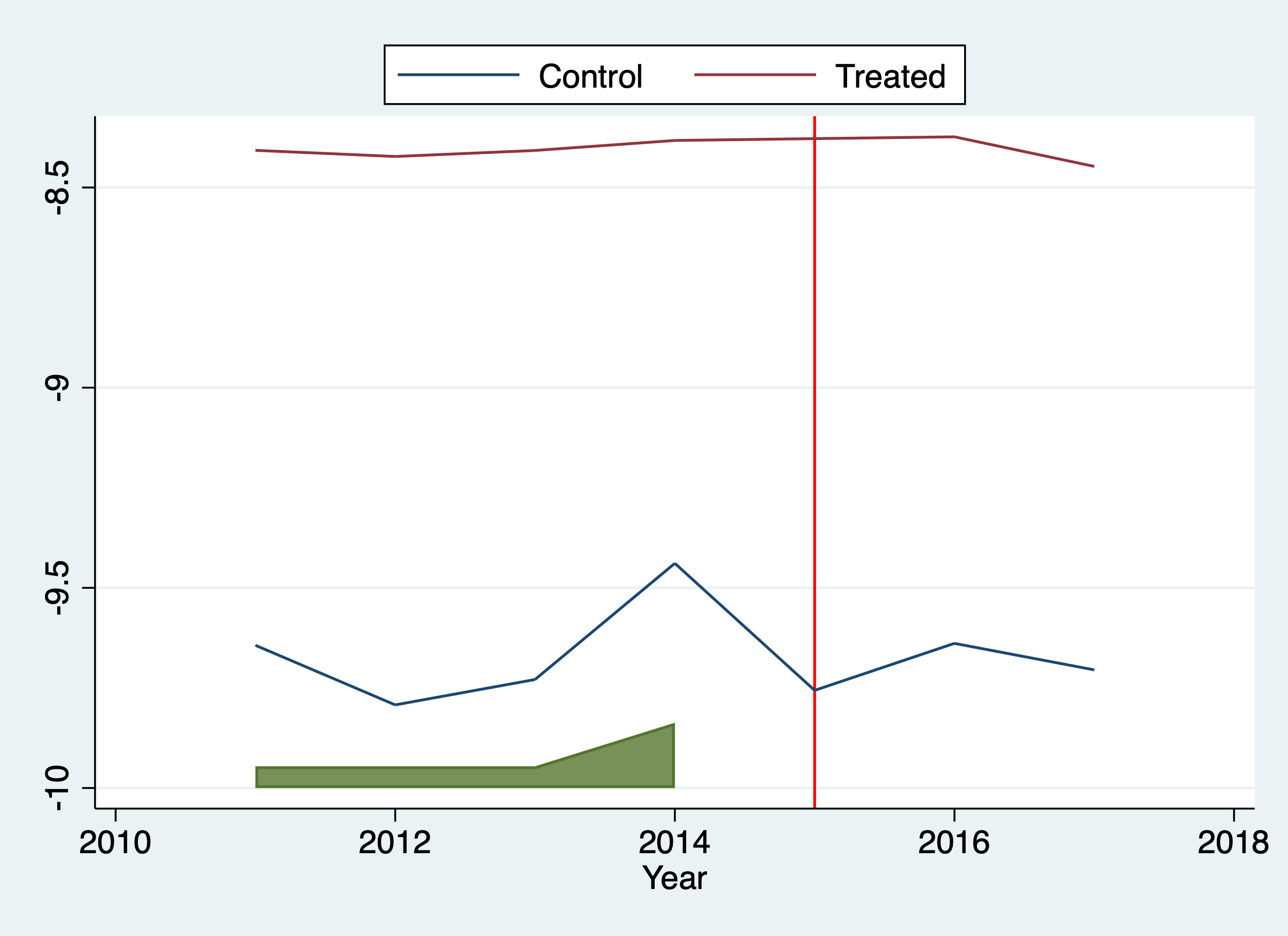

Supplement: S19 Fig — (TIF) [file pone.0285863.s021.tif]

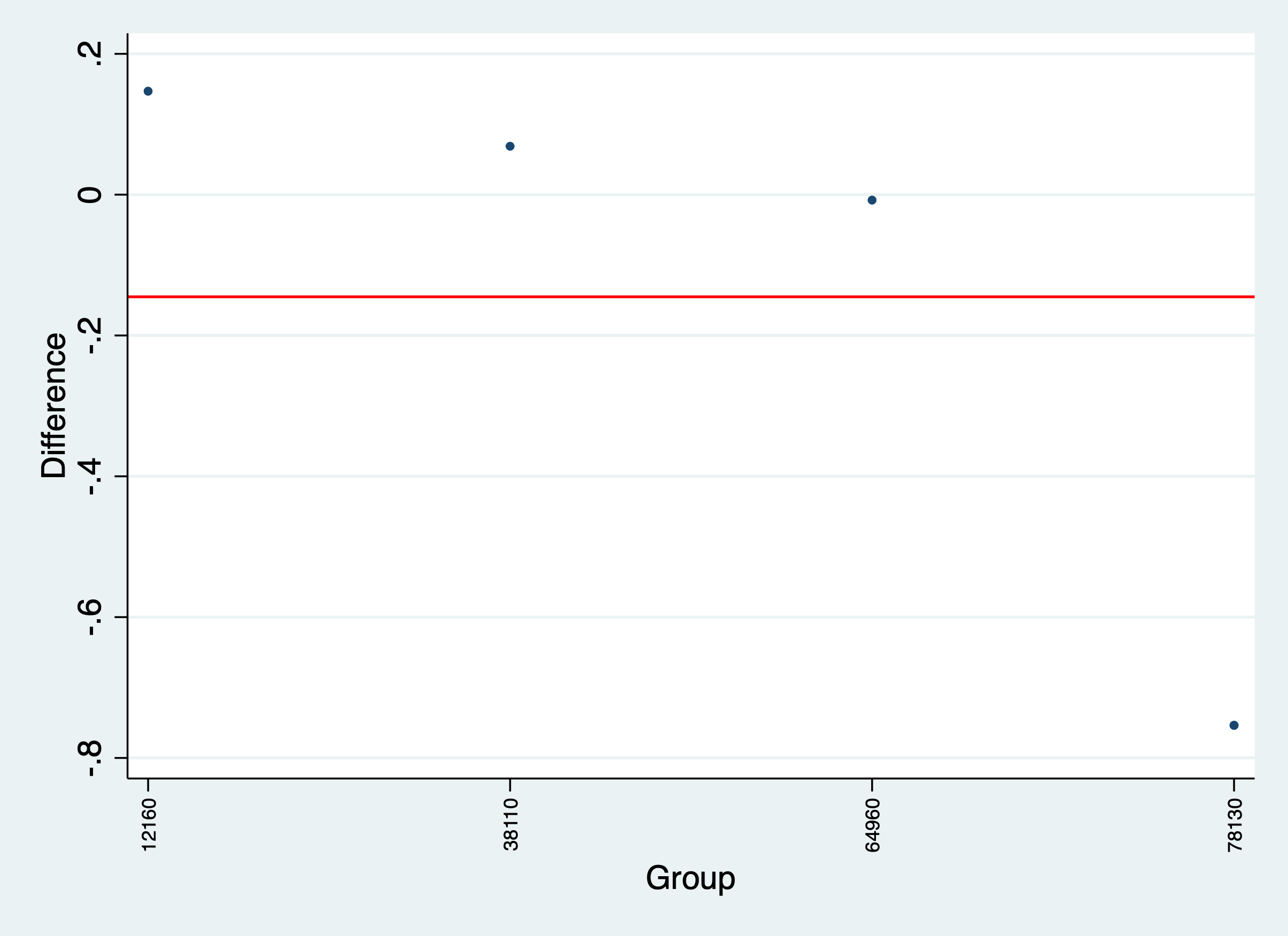

Supplement: S20 Fig — (TIF) [file pone.0285863.s022.tif]

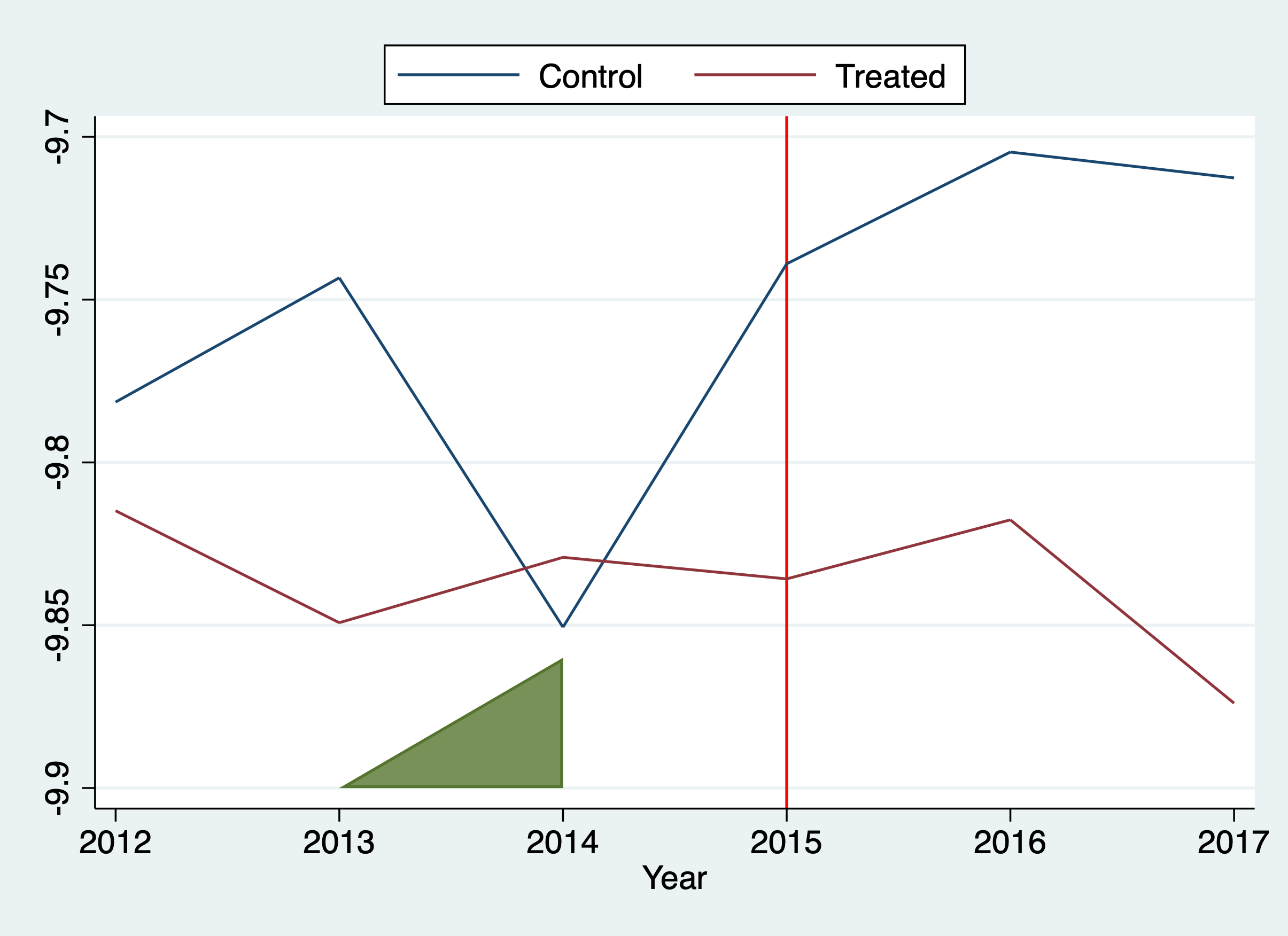

Supplement: S21 Fig — (TIF) [file pone.0285863.s023.tif]

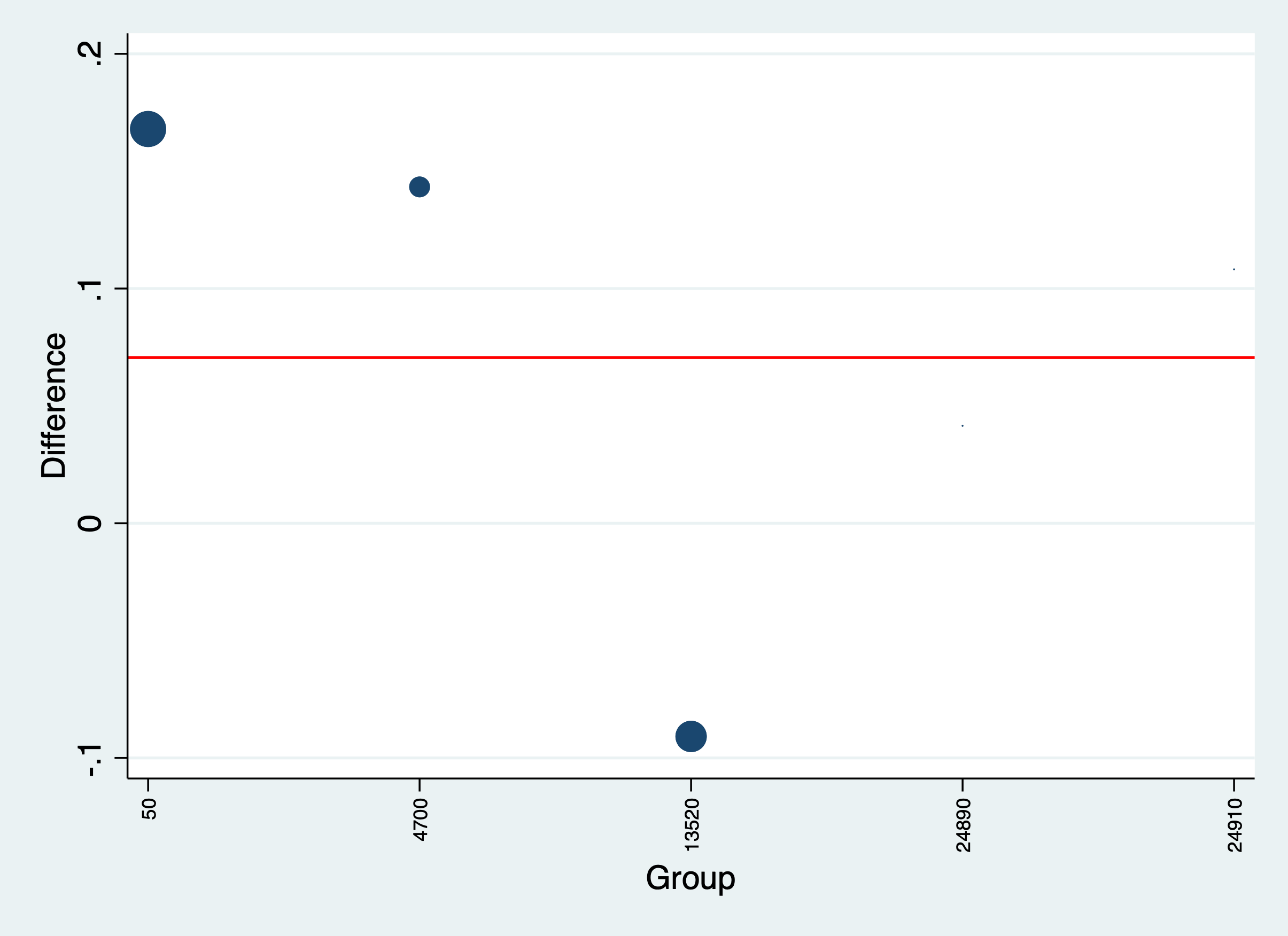

Supplement: S22 Fig — (TIF) [file pone.0285863.s024.tif]

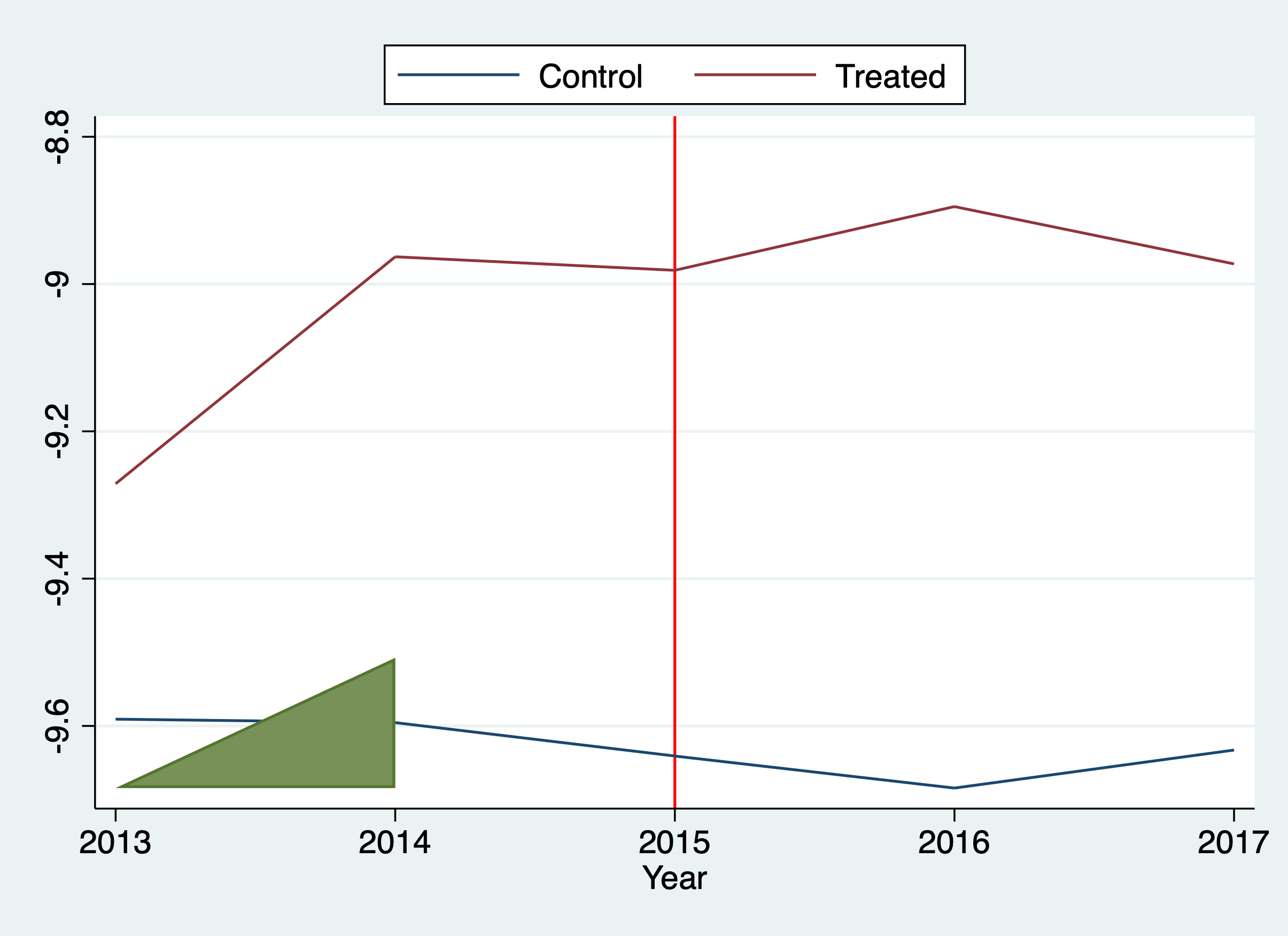

Supplement: S23 Fig — (TIF) [file pone.0285863.s025.tif]

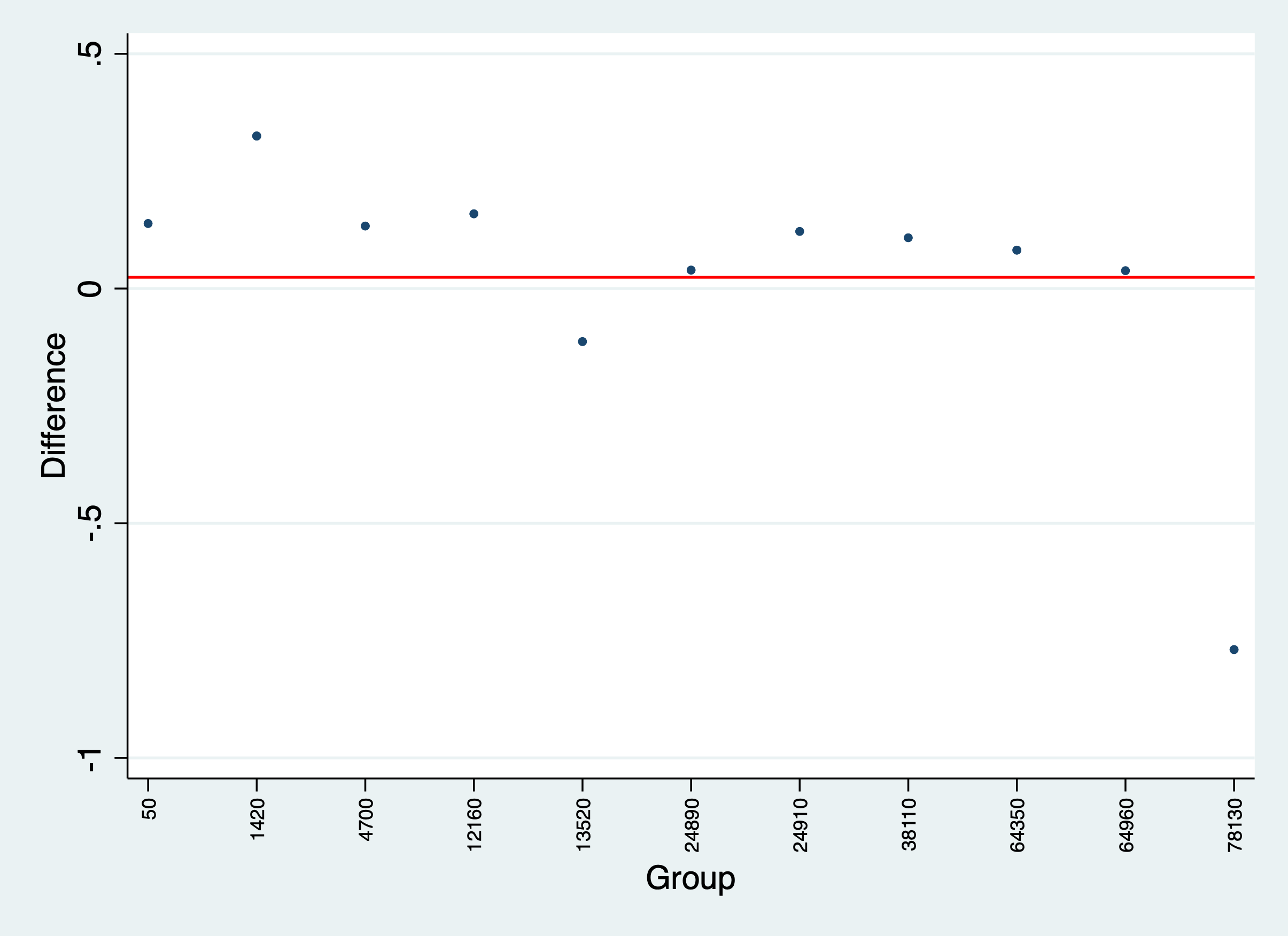

Supplement: S24 Fig — (TIF) [file pone.0285863.s026.tif]

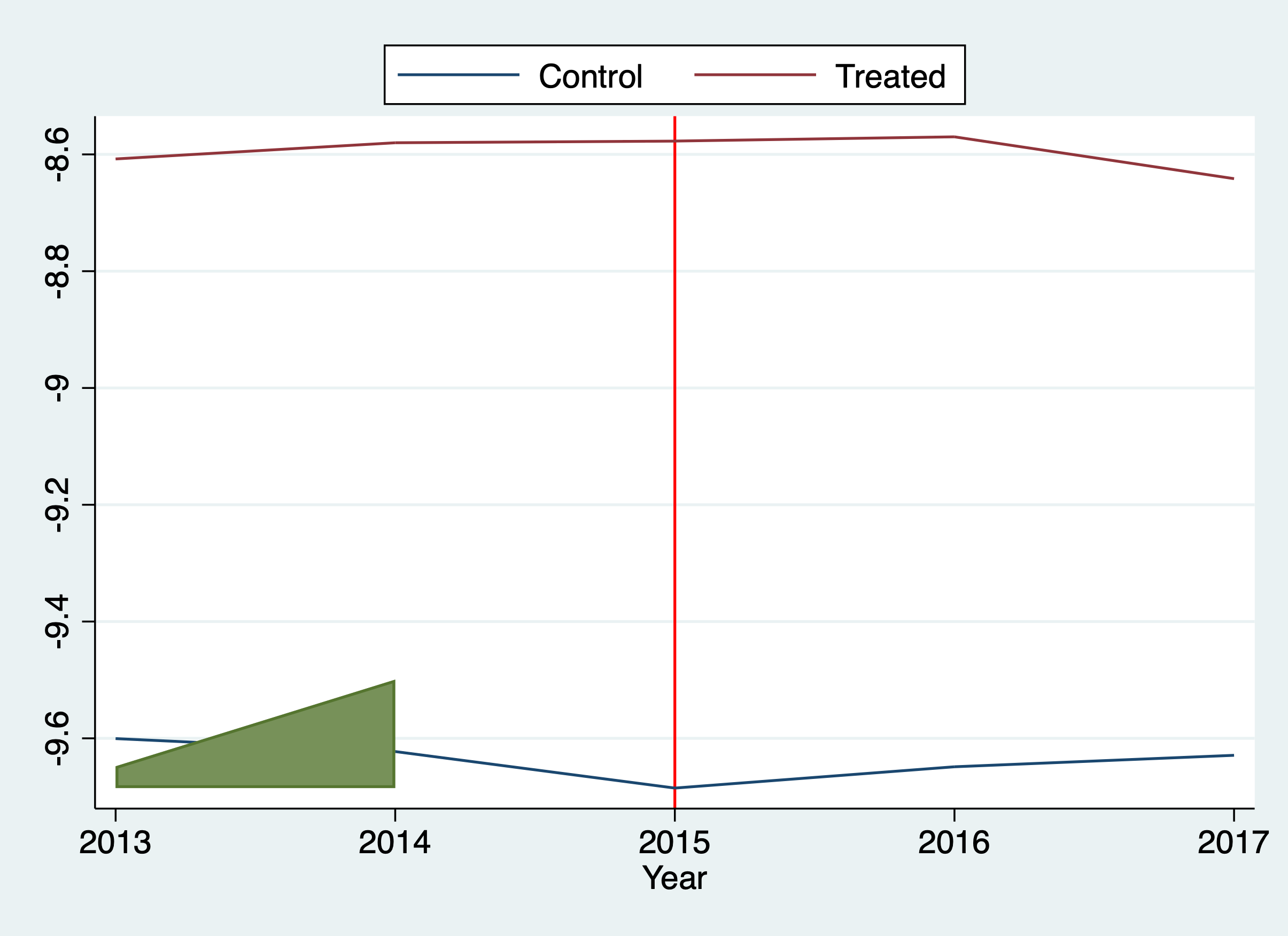

Supplement: S25 Fig — (TIF) [file pone.0285863.s027.tif]

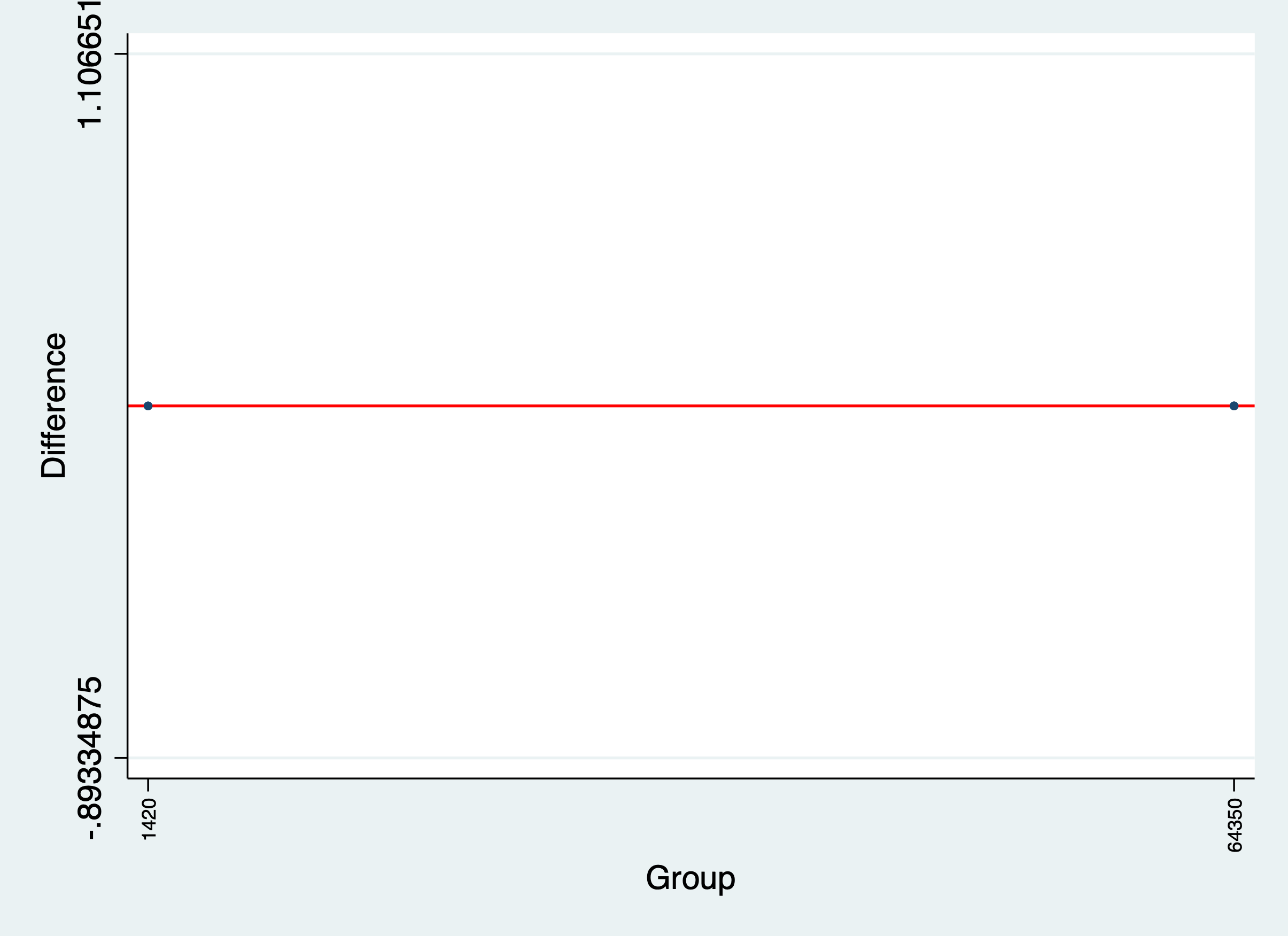

Supplement: S26 Fig — (TIF) [file pone.0285863.s028.tif]

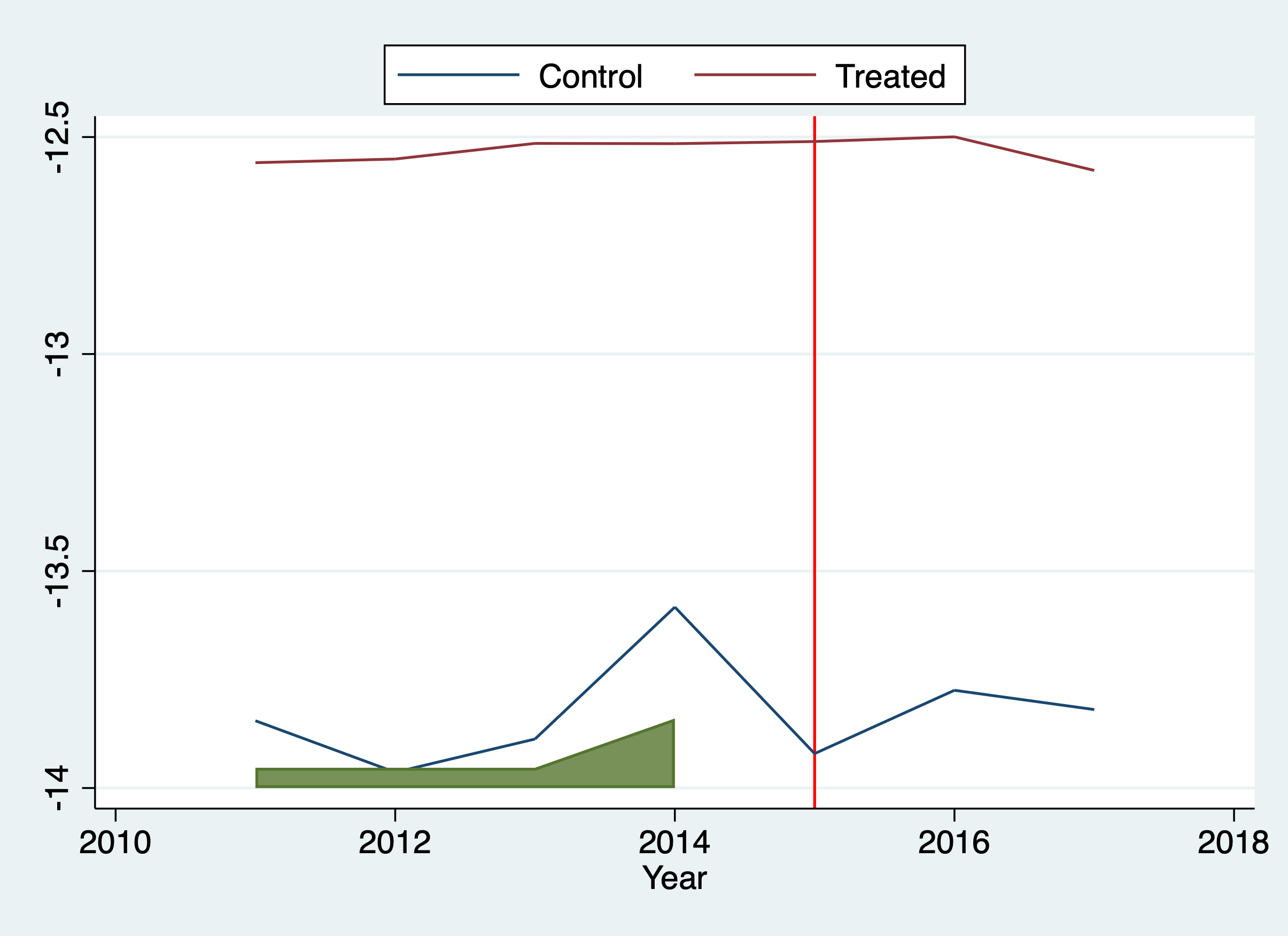

Supplement: S27 Fig — (TIF) [file pone.0285863.s029.tif]

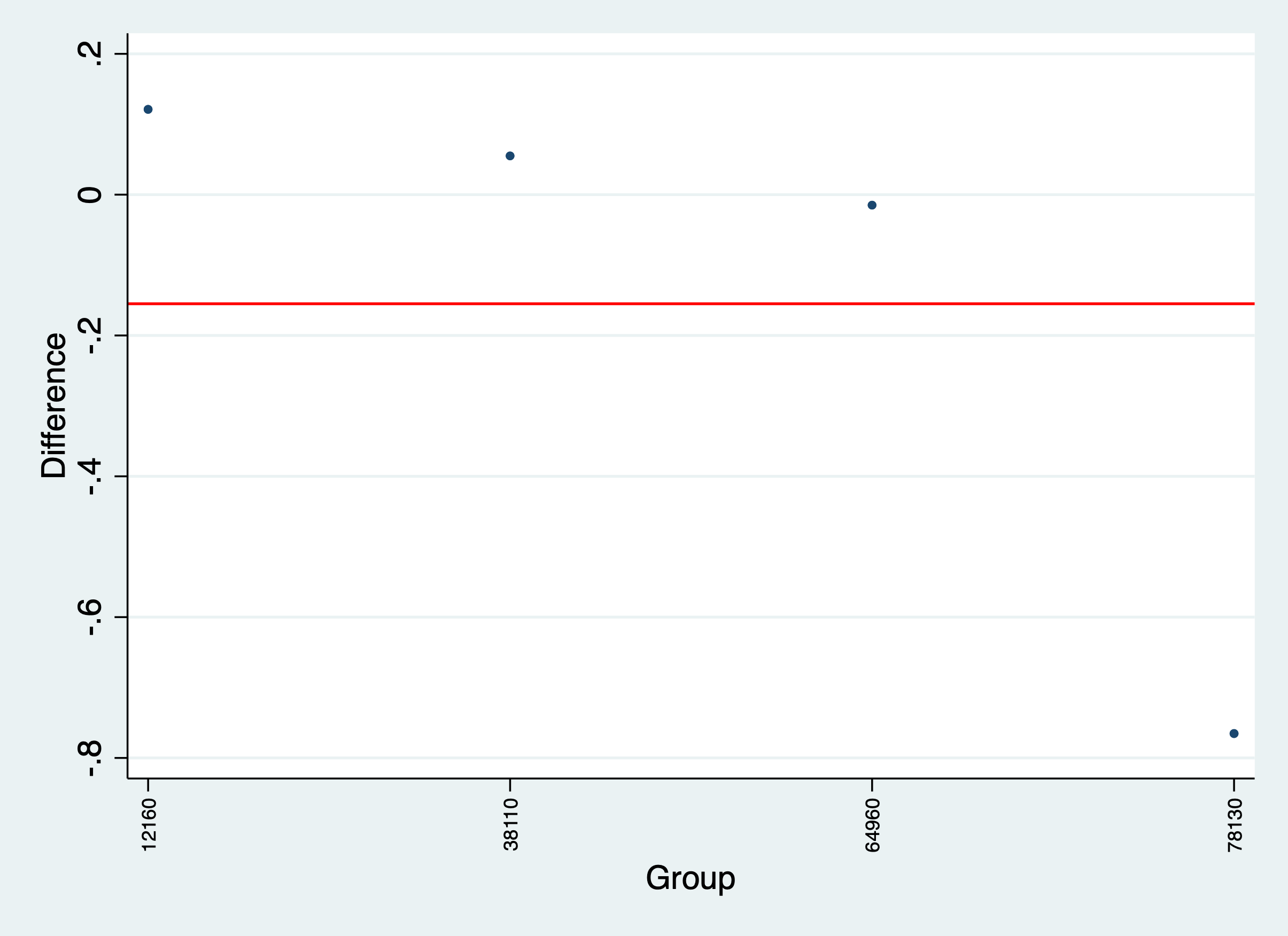

Supplement: S28 Fig — (TIF) [file pone.0285863.s030.tif]

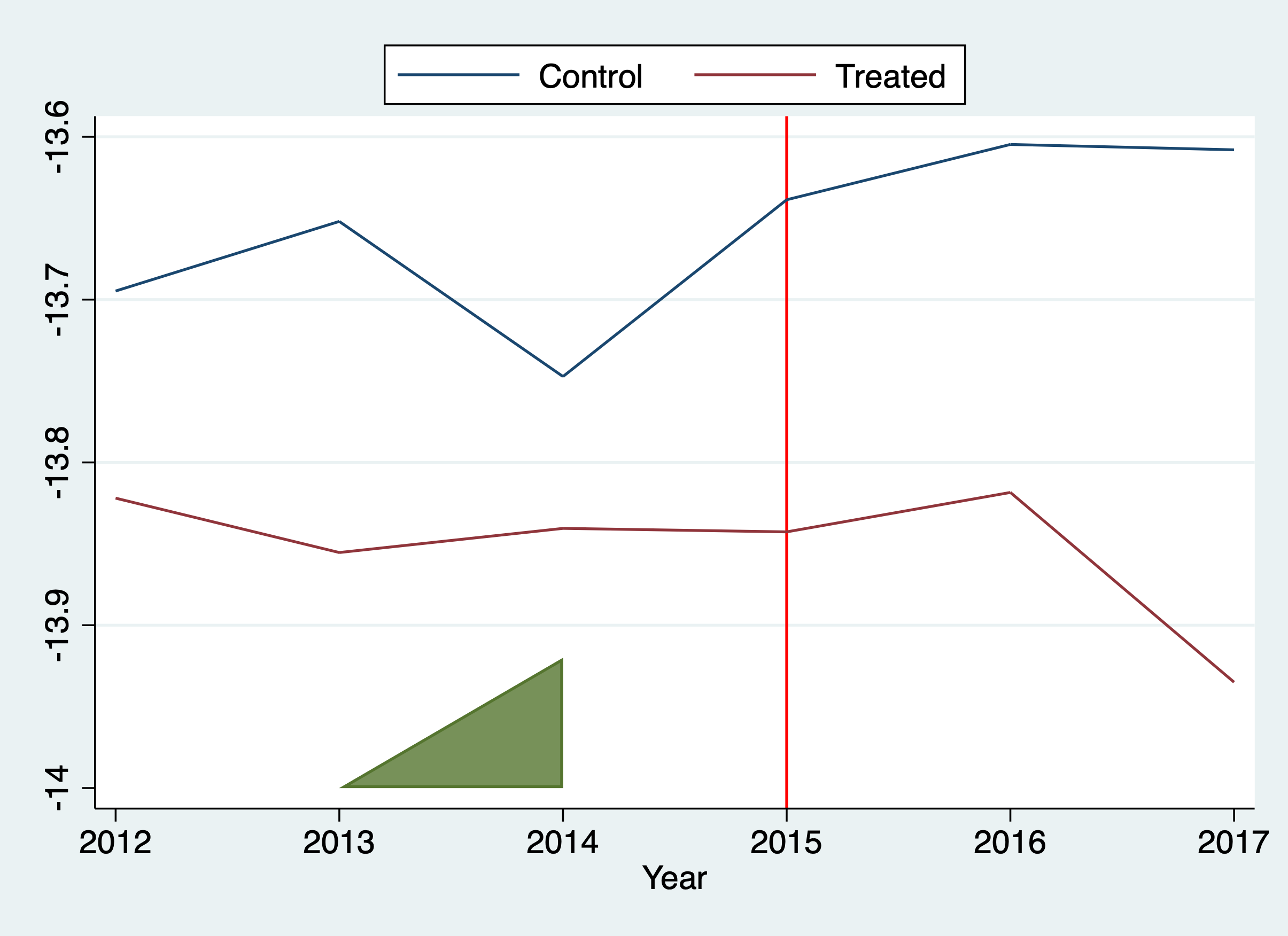

Supplement: S29 Fig — (TIF) [file pone.0285863.s031.tif]

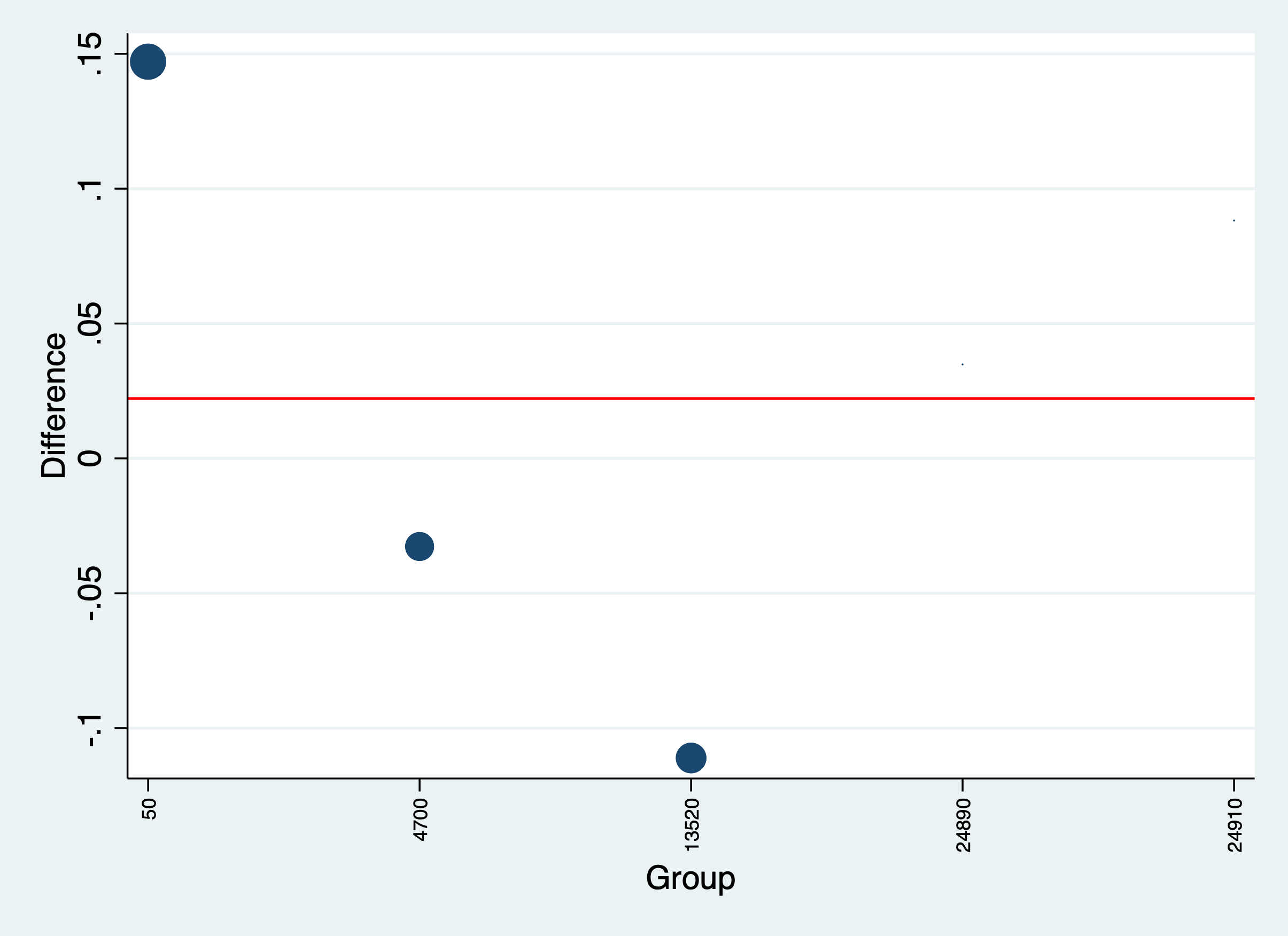

Supplement: S30 Fig — (TIF) [file pone.0285863.s032.tif]

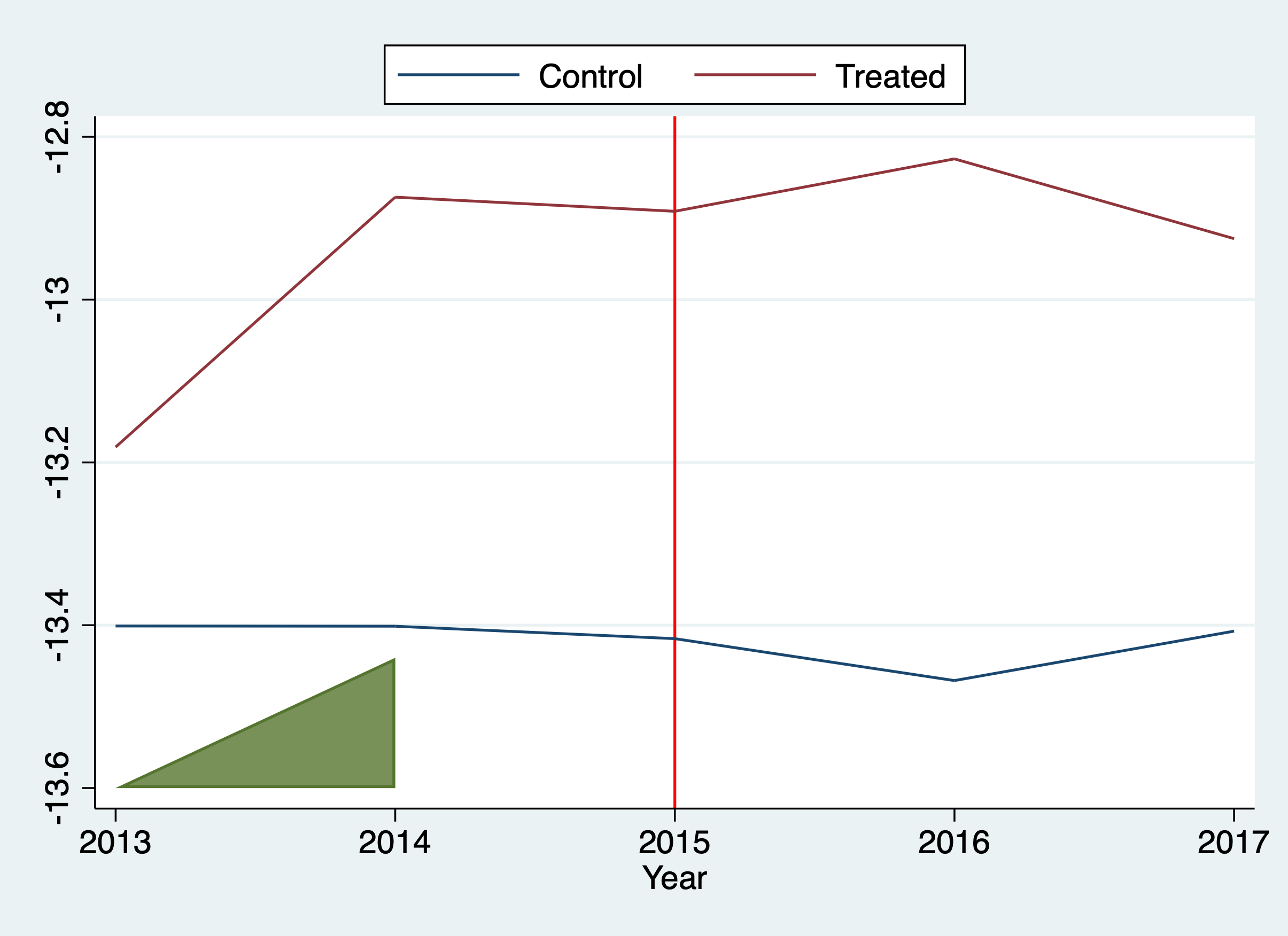

Supplement: S31 Fig — (TIF) [file pone.0285863.s033.tif]

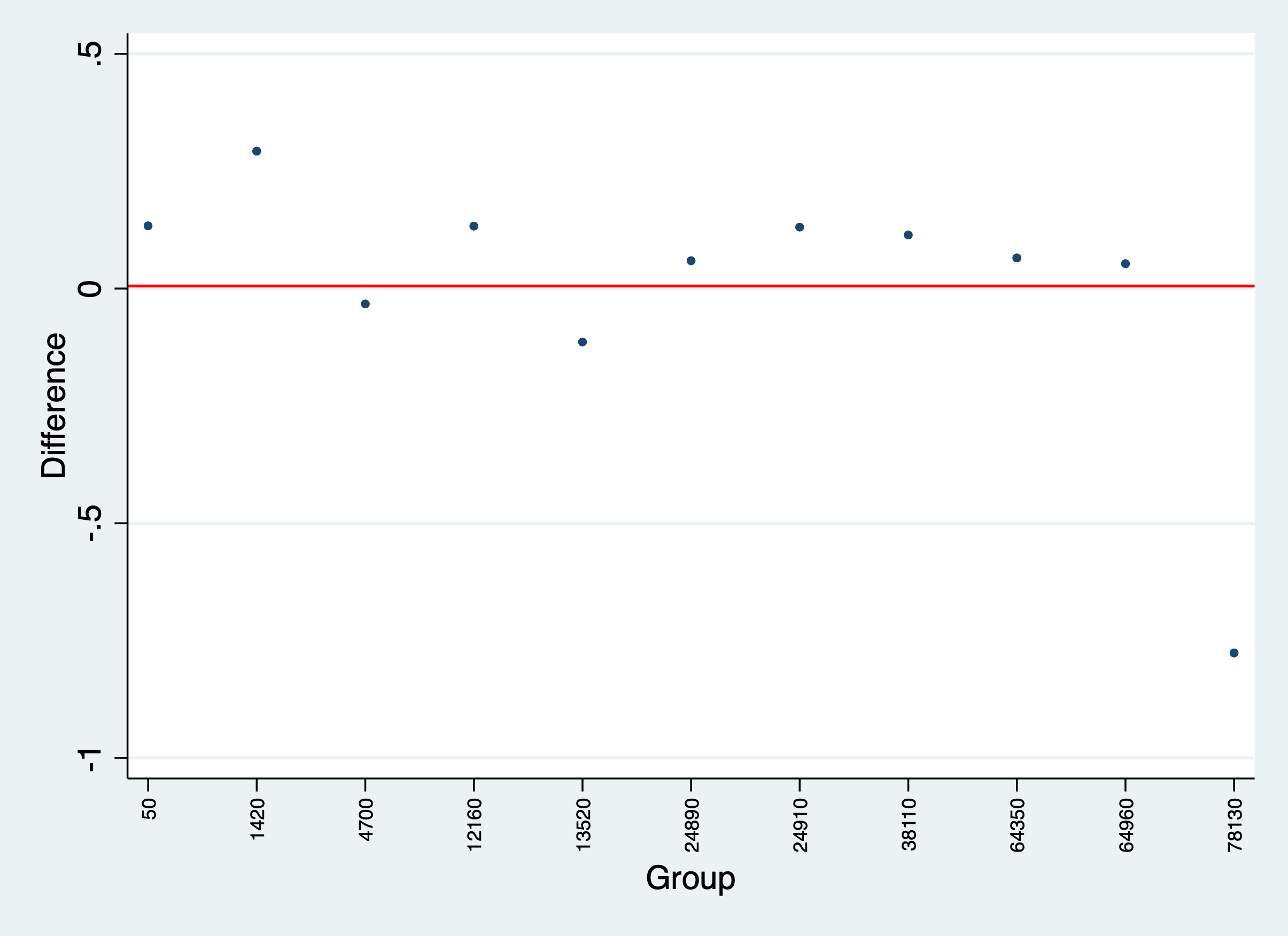

Supplement: S32 Fig — (TIF) [file pone.0285863.s034.tif]

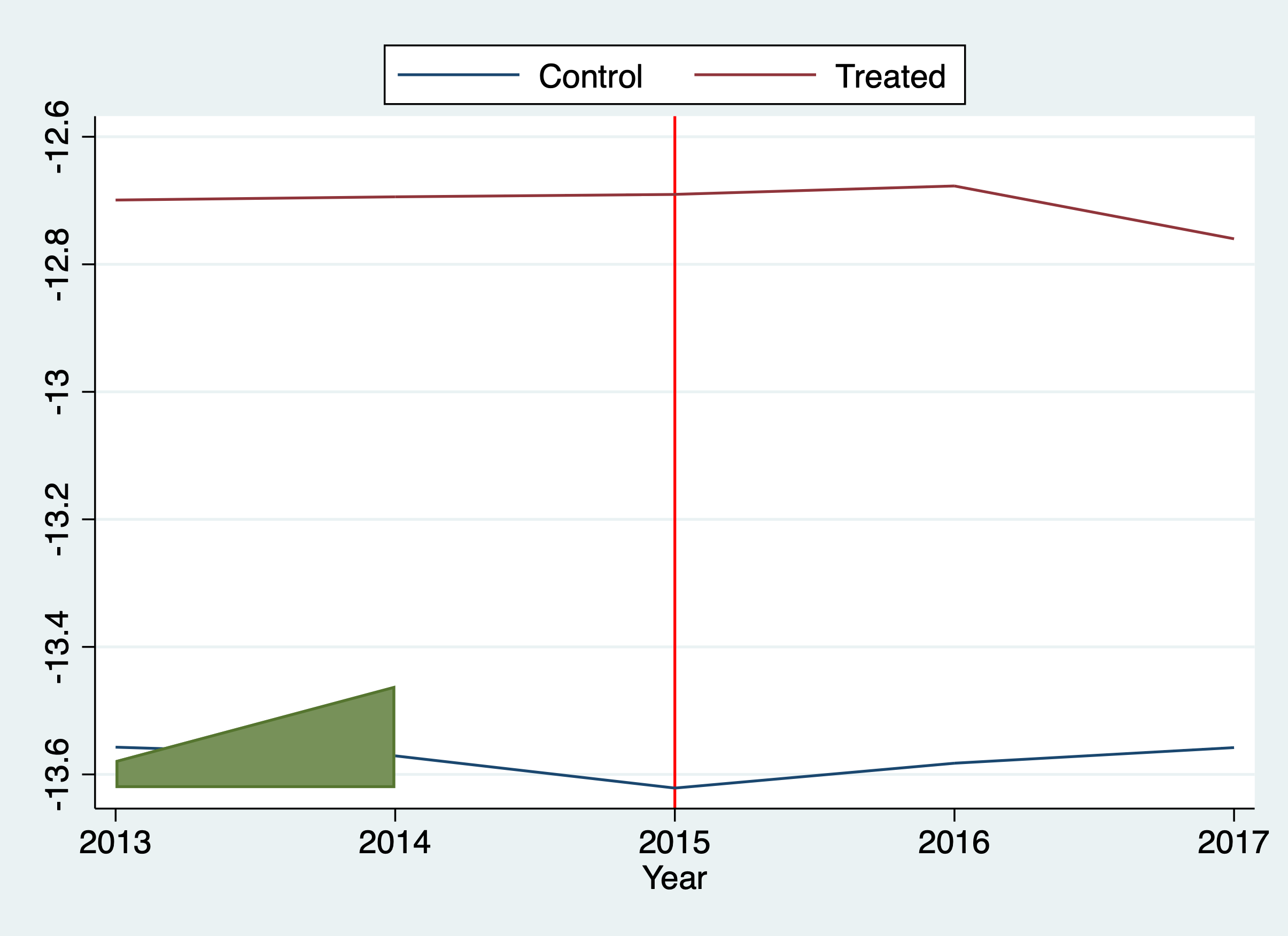

Supplement: S33 Fig — (TIF) [file pone.0285863.s035.tif]
